# PABPC3 drives ovarian cancer metastasis and drug sensitivity by downregulating CLDN1 expression

**DOI:** 10.1038/s41419-025-08151-5

**Published:** 2025-11-17

**Authors:** Hong Zhang, Yiping Lin, Mintao Ji, Yuhan Guo, Haisheng Liang, Kai Kang, Shuangshuang Lu, Zhisen Zhang, Yinyin Shu, Xiaoni Jin, Wenjuan Gan, Qian Xu, Youguo Chen, Yuhong Wang, Zhe Lei, Lingchuan Guo, Chunlin Shao, Lei Chang

**Affiliations:** 1 The First Affiliated Hospital of Soochow University, School of Radiation Medicine and Protection, State Key Laboratory of Radiation Medicine and Protection, Collaborative Innovation Center of Radiation Medicine of Jiangsu Higher Education Institutions, Suzhou Medical College of Soochow University, Suzhou, China; 2 Department of Obstetrics and Gynecology, The First Affiliated Hospital of Soochow University, Suzhou, China; 3 Institute of Radiation Medicine, Shanghai Medical College, Fudan University, Shanghai, China; 4 The Forth Affiliated Hospital of Soochow University, Suzhou Medical College of Soochow University, Suzhou, China; 5 Department of Pathology, The First Affiliated Hospital of Soochow University, Suzhou, China

**Keywords:** Cancer, Cancer

## Abstract

Ovarian cancer remains one of the most lethal malignancies affecting women, with its high mortality rate primarily attributed to the aggressive metastatic nature of the disease, leading to late-stage diagnoses. The challenges posed by tumor metastasis and treatment resistance significantly complicate disease management and substantially reduce survival rates. Thus, elucidating the mechanisms underlying ovarian cancer metastasis is crucial for developing targeted therapies and improving patient outcomes. In this study, through single-nucleus RNA sequencing and analysis of clinical samples, we identify PABPC3 as a key regulator of ovarian cancer metastasis and patient survival. Functional experiments reveal that *PABPC3* knockdown markedly inhibits ovarian cancer cell proliferation and migration, whereas its overexpression exerts the opposite effects. Furthermore, in vivo models confirm that *PABPC3* overexpression significantly enhances metastatic potential. Mechanistically, *PABPC3* promotes tumor metastasis by modulating the expression of CLDN1, a critical component of tight junctions. *PABPC3* knockdown leads to a significant upregulation of CLDN1, while simultaneous CLDN1 knockdown partially rescues the migration-inhibitory effects induced by *PABPC3* depletion. Additionally, clinical analyses demonstrate that high *PABPC3* expression correlates with shorter overall survival, even among patients receiving chemotherapy. Notably, increased PABPC3 protein levels in metastatic lesions are associated with reduced progression-free survival. In conclusion, this study underscores the pivotal role of PABPC3 in ovarian cancer metastasis and patient prognosis, highlighting it as a potential therapeutic target for improving clinical outcomes.

## Introduction

Ovarian cancer is one of the most prevalent gynecologic cancers, holding the third-highest incidence rate after cervical and endometrial cancers, with a five-year relative survival rate of 49% [1]. Each year, approximately 300,000 new cases and 200,000 deaths are reported globally, posing substantial threat to women’s health and survival [2, 3]. Due to the ovary’s deep location in the pelvic cavity and the absence of prominent early symptoms, 70% of patients are diagnosed at advanced stages, making treatment more challenging and significantly reducing survival rates. For those with advanced ovarian cancer, standard treatment involves cytoreductive surgery to decrease cytoreductive surgery to reduce tumor burden, followed by chemotherapy with carboplatin and paclitaxel, which can effectively control tumor progression [1, 4]. In recent years, the introduction of PARP inhibitors for ovarian cancer treatment has brought hope for improved prognosis [5, 6]. However, the mortality rate remains high due to the disease’s aggressive metastatic nature, with approximately 70% of patients presenting with peritoneal metastasis at the time of surgery [7]. Consequently, there is an urgent need to identify and explore novel targets for combating ovarian cancer metastasis.

Numerous cell adhesion molecules function as tumor suppressors by mediating interactions between cells or with the extracellular matrix, thereby regulating intercellular communication and the cellular environment. Loss of cell-cell adhesion is a key mechanism that promotes metastatic progression [8]. Tight junctions are intercellular adhesion complexes in epithelial and endothelial cells that control paracellular permeability. Claudins, a family of transmembrane proteins, are believed to form ion-selective paracellular pores, regulating the paracellular diffusion barrier [9]. Evidence indicates that the Claudin family is closely associated with the EMT process, suggesting these proteins may play a critical role in cancer metastasis [10–12]. Additionally, silencing the CLDN3 gene via siRNA significantly has been shown to inhibit ovarian cancer growth and metastasis [13]. Thus, targeting Claudin family represents a promising therapeutic strategy for limiting ovarian cancer metastasis.

Poly(A)-binding proteins (PABPs) are a conserved family of proteins found in both yeast and humans, known for binding to the poly(A) tails of eukaryotic mRNAs [14]. PABPs are multifunctional proteins regulators involved in various aspects of mRNA homeostasis, including polyadenylation, nonsense-mediated decay, stress responses, control of mRNA translation initiation, and mRNA quality surveillance. Most research has focused on PABP1, which plays a well-characterized role in promoting translation initiation through interactions with translation initiation factors bound to the 5′ end of the mRNA [15]. Meanwhile, previous studies have shown that PABPC3 can drive tumor progression in osteosarcoma, although its role in tumor metastasis remains unclear [16].

In this study, using a combination of single-nucleus RNA sequencing (snRNA seq) and public data analysis, we found that *PABPC3* expression is significantly elevated in metastatic lesions compared to primary tumors, suggesting a potential role for *PABPC3* in the metastatic process. Moreover, both in vitro and in vivo experiments demonstrated that high *PABPC3* expression enhances tumor cell migration, compromises tight junction integrity, and decreases drug sensitivity, highlighting PABPC3 as an essential factor in both metastasis and drug sensitivity.

## Materials and Methods

### Cell culture

The human ovarian cancer SKOV3 cell line and OVCAR3 cell line were purchased from Procell Life Science & Technology Co., Ltd. (Wuhan, China), and the ovarian cancer ID8 cell line was purchased from Chinese Academy of Sciences Type Culture Preservation Center (Shanghai, China). The HEK293T cell line was obtained from ATCC. SKOV3 cells were cultured in McCoy’s 5A medium (Procell) supplemented with 10% fetal bovine serum (FBS, Excell). OVCAR3 was cultured in RPMI 1640 medium (VivaCell) supplemented with 20% FBS (Excell) and 10 µg/ml insulin (Solarbio). ID8, A2780, and HEK293T were cultured in high-glucose DMEM supplemented with 10% FBS (Excell). All media contained 100 ng/mL streptomycin and 100 U/mL penicillin. All cell lines were incubated at 37 °C in a humidified atmosphere containing 5% CO_2_.

### Clinical samples and ethics statements

From each of 20 patients diagnosed with high-grade serous ovarian cancer at the First Affiliated Hospital of Soochow University, we collected paired primary ovarian tumor and matched omental metastasis samples. These samples were used for IHC in Fig. 2G, H. In addition, omental metastasis tumor samples and paired progression-free survival information were collected from 66 patients who were diagnosed with ovarian cancer at the First Affiliated Hospital of Soochow University, which were used in Fig. 6D–F. These cases included 61 high-grade serous ovarian carcinomas (HGSOC), 2 poorly differentiated endometrioid ovarian carcinomas, and 3 well-moderately differentiated ovarian mucinous carcinomas (Detailed pathological subtypes and corresponding PFS information are provided in the Supplementary Table 1). The 66 omental metastasis tumor samples were divided into two groups on the basis of PABPC3 protein levels, then the progression-free survival was calculated in these two groups. The written informed consent was obtained from all patients for research purposes. Patient collection and usage were approved by the First Affiliated Hospital of Soochow University ethics committee and complied with all relevant ethical regulations.

### Western blot

Logarithmic-phase cells were collected and lysed using HPPO buffer containing phenylmethylsulfonyl fluoride (Roche) and protease inhibitors (Roche). The cells were sonicated to obtain total protein. The total protein was mixed with SDS sample buffer, boiled, and separated via SDS-PAGE, then transferred onto a PVDF membrane (Millipore). The membrane was blocked with PBST containing 5% non-fat milk, incubated first with primary antibodies. The antibodies were as follows: anti-Flag (Sigma-Aldrich, F1804-1MG), anti-GAPDH (Millipore, #3241215), and then incubated with HRP-conjugated secondary antibodies (Abbkine, A25022, A25012). After incubation, freshly prepared ECL (NCM Biotech, P10300) solution was added, and the membrane was exposed in a darkroom. The methodology employed in this study was adapted from previous work [17].

### Hematoxylin and eosin (H&E) staining and immunohistochemistry (IHC)

After paraffin-embedded tissue sections were prepared, they were deparaffinized and rehydrated. According to the manufacturer’s instructions, the sections were stained with hematoxylin and eosin (H&E) staining reagent (Solarbio, Beijing, China), and stained tissue sections were observed under a light microscope. For immunohistochemistry (IHC) staining, antigen retrieval was performed using sodium citrate. The methodology employed in this study was adapted from previous work [18]. The sections were treated with 3% hydrogen peroxide to quench endogenous peroxidase activity and blocked with 10% BSA for 1 h. After incubation with the primary antibody overnight at 4 °C. The antibodies were as follows: anti-PABPC3 (Proteintech, 12625-2-AP), anti-CLDN1 (Zenbio, 680135), the sections were incubated with the secondary antibody (AiFang Biological, AFIHC001) for 1 h. DAB (Proteintech, PK10006) was used for color development, and IHC images were captured using a light microscope.

### Co-IP assay

The cells were plated and transfected with the indicated plasmids, and harvested 48 h post-transfection. Cells were lysed by sonication in HPPO buffer. Lysates were cleared by centrifugation at 12,000 rpm for 15 min at 4 °C. Extracts were incubated with anti-FlagM2 antibody (Sigma Aldrich) at 4 °C for 1 h, then incubated with protein G PLUS-Agarose beads (Santa Cruz) at 4 °C overnight. Immunocomplexes were then washed with cold lysis buffer three times, resuspended in SDS sample buffer, incubated at 95 °C for 5 min, and subjected to SDS-PAGE and western blot analysis. The methodology employed in this study was adapted from previous work [17, 19].

### TCGA data analysis

The RNA-seq raw data and clinical survival data of the OV TCGA project in Fig. 2F were downloaded from UCSC Xena (https://xenabrowser.net/hub/). In brief, 427 OV patient data were included in our analyses. For the survival analysis in Fig. 2E, GEPIA2 [20] (http://gepia2.cancer-pku.cn/#index) was used to analyze the relationship between *PABPC3* and prognosis. The survival analysis for the relationship between *PABPC3* and prognosis on the basis of chemotherapy was calculated by the Kaplan–Meier Plotter (https://kmplot.com/analysis/).

### Single-nucleus RNA-seq analysis

We performed snRNA seq from omentum metastasis of HGSOC, and integrated with data from 18 patients [21, 22]. It contained 5 non-malignant ovarian samples, 7 HGSOC tumors, and 7 ovarian cancer omentum metastasis samples of two datasets, including GSE184880 and GSE147082. Among the metastatic samples, 4 were HGSOC omental metastasis samples and 2 were from patients without a confirmed diagnosis of HGSOC. We also generated one additional omental metastasis sample in this study to increase the sample size. After processing by Cell Ranger, the sequencing data were analyzed downstream using Seurat [23]. All the above samples were integrated and used together for the single-cell analysis. The methodology employed in this study was adapted from previous work [24]. First, quality control and normalization were performed on the data to remove low-quality cells and low gene expression noise. Subsequently, high-variance gene selection was conducted, followed by dimensionality reduction analysis (t-SNE and UMAP) to visualize the heterogeneity among cell populations. Differential expression analysis was performed for each cell population to identify population-specific marker genes. Functional enrichment analysis was conducted using databases such as Gene Ontology (GO) to explore the enrichment of biological processes and signaling pathways. Next, GSEA was employed to assess the activity levels of various biological pathways in each cell population, calculating enrichment scores for specific gene sets to quantify pathway activity. Cell communication analysis tools (such as CellChat [25]) were used to explore communication patterns between cell populations, providing insights into intercellular signaling and functional cooperation. The inferCNV (https://github.com/broadinstitute/inferCNV) was used to distinguish the tumors from the epithelial cells on the basis of Normal group.

### Additional experimental methods are provided in the Supplementary Materials due to space constraints

#### Statistics

All data statistical analyses were performed using Excel. Differences between groups were analyzed using Student’s *t*-test or one-way analysis of variance. For data that did not follow a normal distribution, the Mann–Whitney *U* test was used for comparison. The significant level was set at two-tailed *p* < 0.05. Survival analysis was conducted using Kaplan–Meier curves, with the Log-rank test used to assess differences in survival rates. Correlation analysis was performed using Pearson correlation coefficients. All tests were two-tailed, with *p* < 0.05 considered statistically significant.

## Result

### The characteristics of ovarian cancer microenvironments

**Fig. 1:**
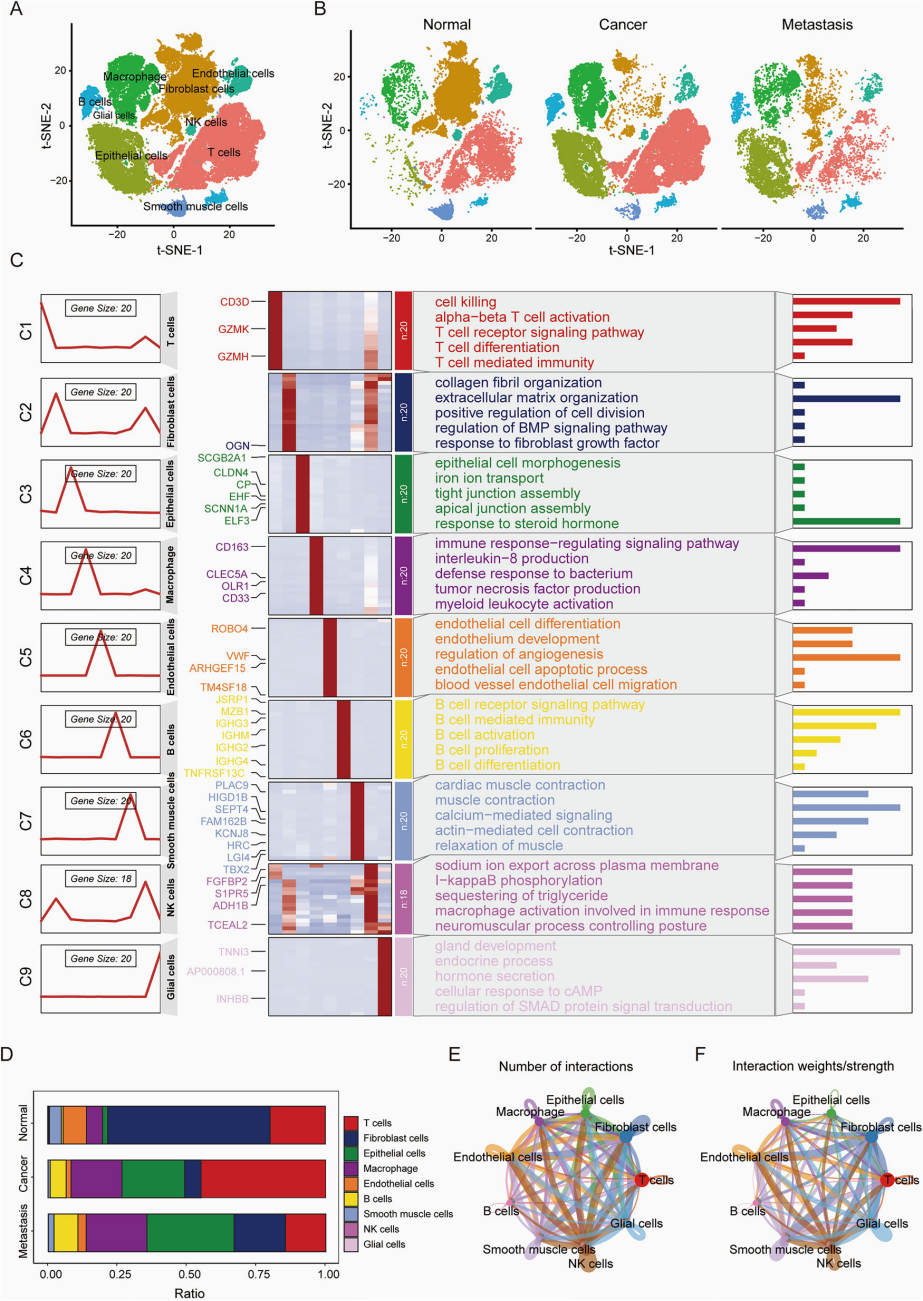
Characteristics of the ovarian cancer microenvironment. **A** The t-SNE plot illustrates the distribution of major cell types within the ovarian cancer dataset, encompassing 70,508 cells across nine distinct cell types, including epithelial cells, fibroblasts, endothelial cells, and T cells. **B** The t-SNE plot displays the distribution and abundance of primary cell types in normal tissue, primary ovarian tumors, and metastatic tumors. **C** Gene Ontology (GO) enrichment analysis highlights the key biological pathways enriched in each cell type. **D** Comparative analysis of the proportions of different cell types across the three groups. **E**, **F** Cell-Chat analysis reveals the number and strength of interactions between various cell types.

High-grade serous ovarian cancer (HGSOC) is among the most aggressive gynecological malignancies. To systematically explore intratumoral heterogeneity and metastasis in ovarian cancer, we conducted sn-RNA sequencing on omental metastasis of HGSOC and integrated these results with data from 18 patients [21, 22] (5 non-malignant ovarian samples, 7 HGSOC tumors, and 6 ovarian cancer omentum metastasis samples). We analyzed 39150 features across 70508 cells and identified nine distinct cell clusters, including epithelial cells, fibroblast cells, endothelial cells, macrophage, B cells, glial cells, smooth muscle cells, NK cells, and T cells (Fig. 1A). We then performed signaling enrichment analysis for each cell type and examined the heterogeneity of the tumor microenvironment. Our finding reveals substantial differences in the microenvironments of primary tumors versus omental metastases. As shown in the UMAP plot (Fig. 1B), distinct subpopulations can be observed. Furthermore, we observed that the T cells were enriched in pathways related to cytotoxicity and T cell-mediated immunity, while macrophages, expressing high levels of CD63, CD33, and OLR1, are enriched in pathways associated with immune response regulation and leukocyte activation (Fig. 1C). Consistently, the compositional analysis indicates that macrophages and T cells are significantly more abundant in primary tumors but reduced in metastatic tumors, whereas fibroblast cells show the opposite trend. (Fig. 1D) These findings underscore the distinct microenvironments of primary tumors and omental metastases.

We then conducted cellular communication analysis to elucidate interactions between different subpopulations. Our analysis is about two aspects, the number and strength of cell-to-cell interactions. The number of interactions may help to identify potential signaling hubs. We suggest that cell clusters with higher interaction counts can serve more important roles as communication relays. Regarding the second aspect, the strength of cell-to-cell interactions, the clusters with high interaction strength may occupy critical paths at the center of the regulatory network. The results showed that fibroblast cells, endothelial cells, and NK cells were the most active in terms of cellular interactions, displaying frequent communication among these groups. In contrast, B cells and T cells exhibited minimal interaction (Fig. 1E, F), likely due to the release of immunosuppressive factors by tumor cells within the tumor microenvironment, which dampens immune responses. These complex regulatory interactions among various cell types within the tumor microenvironment further highlight the heterogeneity between metastatic and primary lesions.

### *PABPC3* exhibited high expression in metastatic lesions

**Fig. 2:**
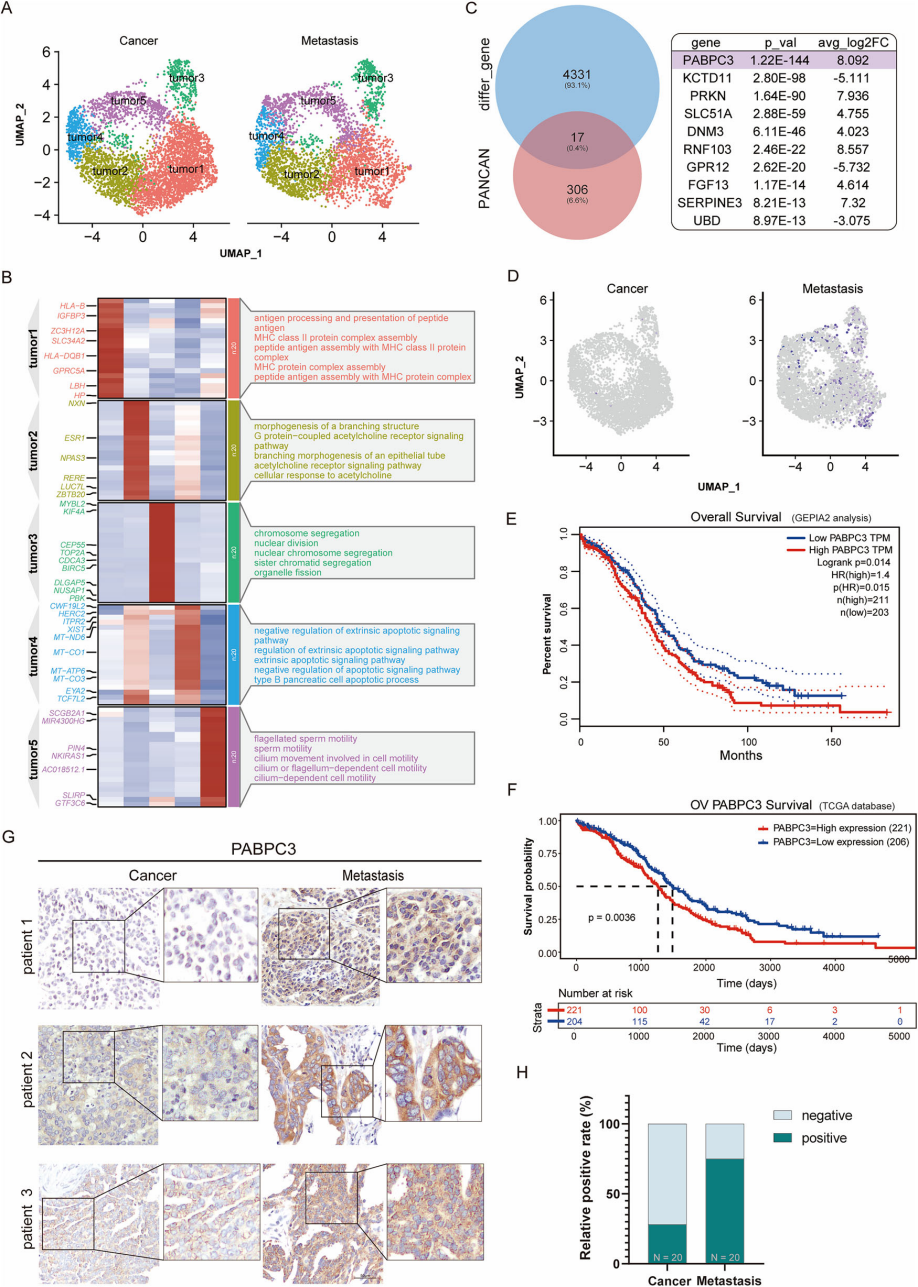
PABPC3 is highly expressed in metastatic lesions. **A** UMAP visualization depicting the distribution of tumor cells in primary and metastatic ovarian tumors. **B** Gene Ontology (GO) enrichment analysis identifying the key biological pathways enriched in different tumor cell clusters. **C** A Venn diagram illustrating the intersection of differentially expressed genes between primary and metastatic ovarian cancer cells with feature genes from the TCGA pan-cancer cohort. **D** UMAP plot showing the expression levels of PABPC3 in tumor cells from primary and metastatic ovarian cancers. **E** Survival analysis using GEPIA demonstrating the impact of PABPC3 expression on overall survival. **F** Survival analysis from the TCGA database further validating the association between PABPC3 expression and overall survival. **G** Immunohistochemical (IHC) staining of primary and metastatic ovarian cancer tissues from patients, with paired samples (left and right) representing tissues from the same patient. **H** Statistical analysis of the proportion of tumors with high PABPC3 expression in primary versus metastatic ovarian cancer. Statistical significance was determined using a two-sided unpaired Student’s *t*-test with a 95% confidence interval. Scale bars, 100 µm.

To further examine differences in epithelial cells between primary and metastatic tumors, we subclustered the epithelial cells. Although there are individual differences among ovarian cancer patients, our study specifically focuses on the factors driving ovarian cancer metastasis at the population level. Therefore, we conducted a combined analysis of single-cell data. First, using CNV scores to assist in identifying malignant cells, we classified epithelial cells into primary and metastatic tumor groups (Fig. S1A). which provided a basis for further cell dimension reduction analysis and cluster analysis. These cells were further clustered and visualized using UMAP, revealing five subpopulations: Tumor 1 was mainly enriched in peptide processing and protein complex assembly; Tumor 2 in acetylcholine-related signaling pathways; Tumor 3 in chromatin separation and nuclear division; Tumor 4 in apoptosis-related pathways; and Tumor 5 in ciliary movement. Notably, there were no significant differences in cell numbers between the primary and metastatic tumor groups (Fig. 2A, B).

Next, we analyzed gene expression differences in tumor cells between primary and metastatic ovarian cancers, identifying 4348 differentially expressed genes (*p*_val_adj < 0.01, avg_log2FC > 3 & avg_log2FC < −3). By integrating data from the TCGA Pan-Cancer cohort, we identified 17 signature genes significantly correlated with overall survival (*p* < 0.01), with *PABPC3* showing the most substantial difference (Fig. 2C). *PABPC3* was highly expressed in metastatic tumors, with much lower expression in primary tumors, suggesting that *PABPC3* is a potential key driver of ovarian cancer metastasis (Fig. 2D). Survival analysis using various databases was performed for PABPC3 and indicated that the higher *PABPC3* expression in ovarian cancer correlates with shorter overall survival (Fig. 2E, F). PABPC3 protein is mainly expressed in the cytosol [26], and in order to assess PABPC3 protein expression levels in patients, we performed immunohistochemistry analysis on clinical samples from primary and metastatic ovarian tumors. We counted samples of primary and metastatic lesions from 20 patients and showed that PABPC3 protein expression was generally elevated in metastatic lesions compared with primary lesions from the same patient (Figs. 2G, H and S5A). It was indicated that PABPC3 may promote ovarian cancer metastasis and could represent a viable therapeutic target.

### *PABPC3* knockdown significantly inhibited cell proliferation and metastasis

**Fig. 3:**
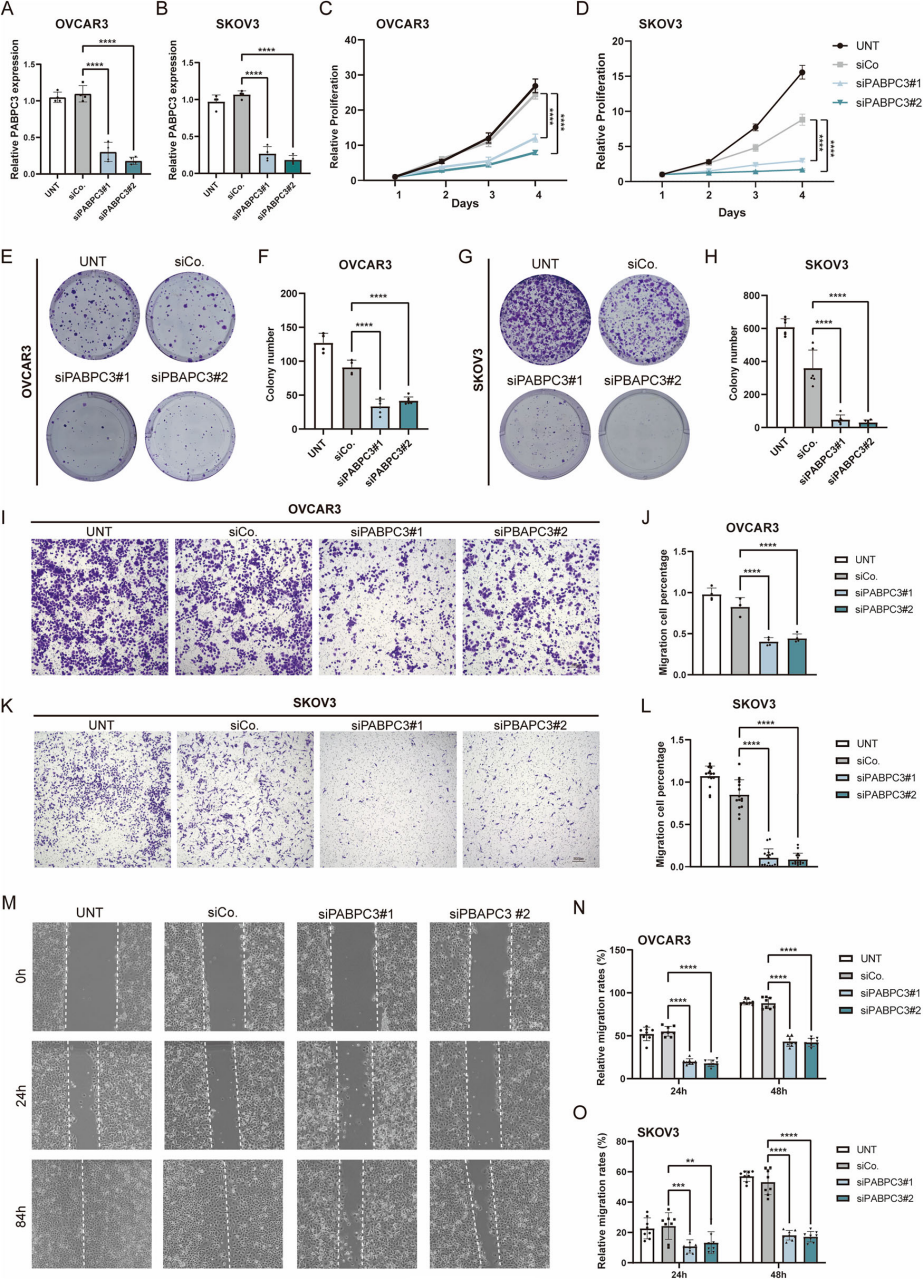
*PABPC3* knockdown inhibits cell proliferation and metastasis. **A**, **B** Relative expression levels of *PABPC3* were quantified by qPCR following transfection with siCo. (control) and siPABPC3 in OVCAR3 and SKOV3 cells. **C**, **D** CCK-8 assay evaluating the proliferation activity of OVCAR3 and SKOV3 cells after transfection. **E–H** Colony formation assay demonstrating the clonogenic survival of OVCAR3 and SKOV3 cells following PABPC3 knockdown. **I** Transwell assay indicating a significant reduction in the migration ability of OVCAR3 cells upon PABPC3 knockdown. **J** Quantification of OVCAR3 cell migration percentage in the Transwell assay post-transfection. **K** Transwell assay indicating a significant reduction in the migration ability of SKOV3 cells upon PABPC3 knockdown. **L** Quantification of SKOV3 cell migration percentage in the Transwell assay post-transfection. **M** Wound healing assay showing impaired migration and reduced wound closure capacity in OVCAR3 cells following PABPC3 knockdown. **N**, **O** Quantification of OVCAR3 and SKOV3 cells migration percentage in the wound healing assay post-transfection. Bar and line charts represent the mean ± SD. Statistical significance was determined using a two-sided unpaired Student’s *t*-test with a 95% confidence interval. Each experiment was conducted independently three times with consistent results. **p < 0.01, ****p < 0.0001. Scale bars, 300 µm.

We previously observed that *PABPC3* is highly expressed in metastatic lesions and is closely associated with overall survival in patients. Here, we further investigated the effects of *PABPC3* on the ovarian cancer cell line OVCAR3 and SKOV3 by siRNA. First, RT-qPCR was performed to validate the interference efficiency of siRNA, showing that the transcriptional levels of *PABPC3* were significantly reduced in the siPABPC3 groups (Fig. 3A, B). Next, we assessed the impact of *PABPC3* knockdown on cell viability and proliferation using CCK8 assay and colony formation assay. Results of CCK8 assay suggested that either in OVCAR3 or SKOV3, knockdown of *PABPC3* significantly reduced the cell viability, resulting in slower cell growth (Fig. 3C, D). Similarly, colony formation assays showed notably smaller and fewer colonies in the *PABPC3* knockdown group compared to controls, indicating a substantial effect on cell proliferation (Fig. 3E–H). To examine the impact on metastatic potential, we conducted Transwell assays, which revealed a significant decrease in the number of migrating cells in the *PABPC3* knockdown group versus controls (*P* < 0.05), suggesting reduced metastasis capacity (Fig. 3I–L). Next, wound-healing assays were used to test the migration ability. This result showed significantly slower wound closure in the *PABPC3* knockdown group compared to the control group (Figs. 3M–O and S1B), further implicating *PABPC3* in promoting metastasis.

### *PABPC3* knockdown impairs metastatic potential via CLDN1

**Fig. 4:**
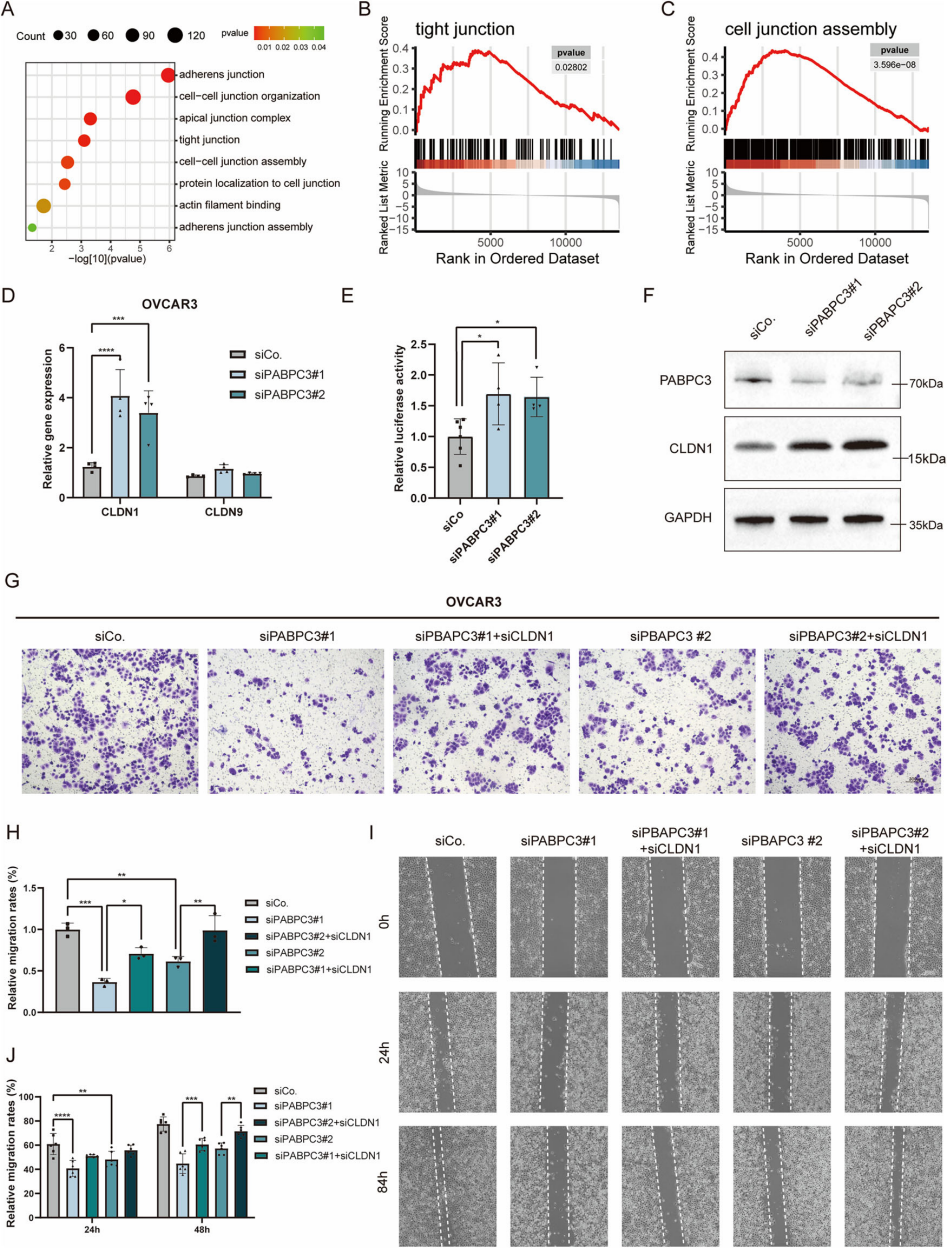
*PABPC3* knockdown regulates cell metastasis capacity via CLDN1. **A** Gene Ontology (GO) enrichment analysis of differentially expressed genes in ovarian cancer cells stratified by *PABPC3* expression levels. **B**, **C** Gene Set Enrichment Analysis (GSEA) identifying pathways associated with tight junctions and cell junction assembly. **D** Real-time qPCR analysis of *CLDN1* and *CLDN9* expression levels following *PABPC3* knockdown. **E** Reporter assay demonstrating that *PABPC3* enhances *CLDN1* promoter activity. **F** Western blot analysis showing increased CLDN1 protein levels upon *PABPC3* knockdown. **G** Transwell assay revealing that simultaneous knockdown of *PABPC3* and *CLDN1* partially rescues the migration-inhibitory effect of *PABPC3* depletion. **H** Quantification of OVCAR3 cell migration percentage in the Transwell assay. **I** Wound healing assay showing enhanced migration ability of OVCAR3 cells following simultaneous *PABPC3* and *CLDN1* knockdown. **J** Quantification of wound healing assay results in OVCAR3 cells. Bar and line charts represent the mean ± SD. Statistical significance was determined using a two-sided unpaired Student’s *t*-test with a 95% confidence interval. Each experiment was independently repeated three times with consistent results. *p < 0.05, **p < 0.01, ***p < 0.001, ****p < 0.0001. Scale bars, 300 µm.

To explore the underlying mechanisms, we categorized tumor cells into PABPC3^low^ and PABPC3^high^ groups and analyzed gene expression profiles and GO signaling pathway alterations. The *PABPC3* transcript levels in each dataset sample were assessed, and the mean expression value was used as a threshold to classify samples into high (above the mean) and low (below the mean) expression groups for further analysis. Pathway analysis revealed significant changes in cell adhesion-related processes, particularly in tight and adherens junctions. GSEA enrichment analysis further demonstrated increased activation of tight junction and cell junction assembly pathways in the PABPC3^low^ group compared to the PABPC3^high^ group (Fig. 4A–C). To determine whether *PABPC3* knockdown influenced cell adhesion, we checked the expression of tight junction-related genes. In SKOV3 cells, knockdown of *PABPC3* led to a significant upregulation of Claudin family members *CLDN1* and *CLDN9* (Fig. S1C, D), suggesting enhanced tight junction integrity. Validation in OVCAR3 cells further confirmed a pronounced increase in *CLDN1* expression (Fig. 4D). To elucidate the regulatory relationship between PABPC3 and CLDN1, we conducted luciferase reporter assays and western blot analysis. The results indicated that *PABPC3* knockdown activated *CLDN1* promoter activity (Fig. 4E) and increased CLDN1 protein levels (Fig. 4F). And the Co-immunoprecipitation (Co-IP) assays demonstrated that CLDN1 had no direct interaction with PABPC3 at the protein level (Fig. S1E), while MKRN3 is a reported interacting protein with PABPC3 [27]. Although Co-IP showed no direct interaction between them, luciferase reporter assays indicated that PABPC3 can modulate CLDN1 promoter activity. It suggests an indirect regulatory mechanism, warranting further investigation.

Next, we performed simultaneous knockdown of *PABPC3* and *CLDN1* in OVCAR3 cells and compared the phenotypic effects with *PABPC3* knockdown alone to assess potential rescue effects. Wound healing and Transwell migration assays demonstrated that *CLDN1* depletion (knockdown efficiency shown in Fig. S1F) partially rescued the impaired migration phenotype induced by *PABPC3* knockdown (Fig. 4G–J), confirming the crucial role of CLDN1 in mediating PABPC3’s function.

Taken together, *PABPC3* knockdown inhibited cell proliferation and metastasis by modulating CLDN1-regulated tight junction integrity.

### *PABPC3* overexpression promoted cell metastasis and reduced the tight junction integrity

**Fig. 5:**
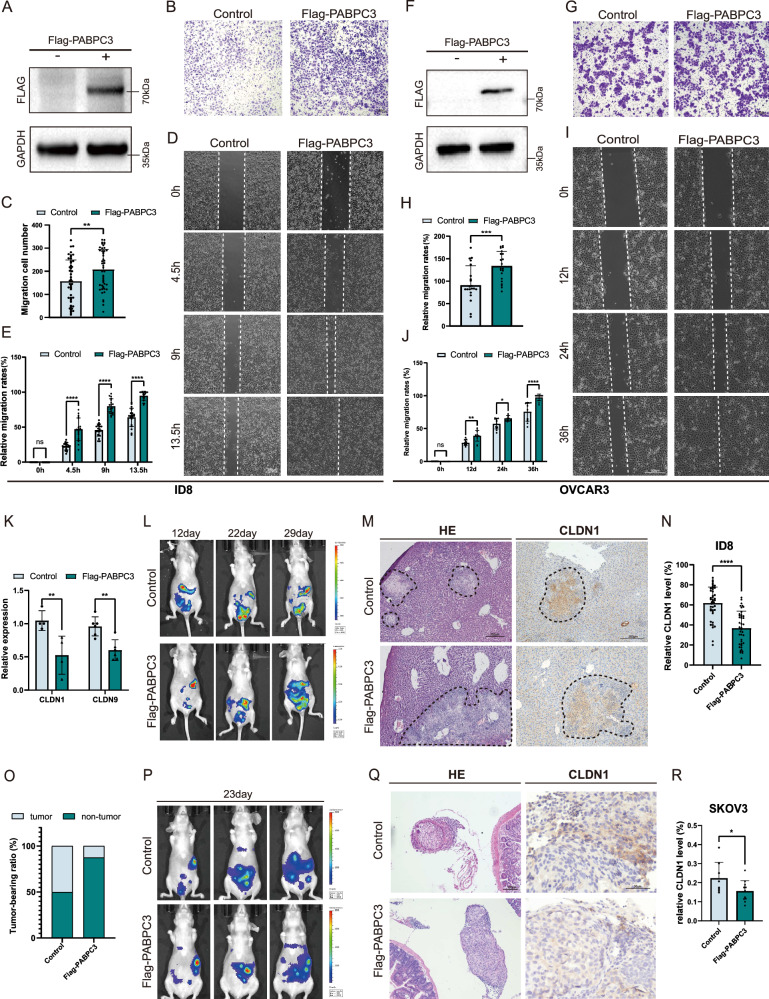
*PABPC3* overexpression promotes cell metastasis and compromises tight junction integrity in vitro and in vivo. **A** An ID8 cell line with stable *PABPC3* overexpression was established, and its expression levels were validated by Western blot. **B** Transwell assay demonstrating significantly enhanced migration of ID8 cells following *PABPC3* overexpression. **C** Quantification of ID8 cell migration percentage in the Transwell assay after *PABPC3* overexpression. **D** Wound healing assay illustrating increased migratory capacity of ID8 cells upon *PABPC3* overexpression. **E** Quantification of wound healing assay results in ID8 cells. **F** An OVCAR3 cell line with stable *PABPC3* overexpression was established, and its expression levels were validated by Western blot. **G** Transwell assay demonstrating significantly enhanced migration of OVCAR3 cells following *PABPC3* overexpression. **H** Quantification of OVCAR3 cell migration percentage in the Transwell assay after *PABPC3* overexpression. **I** Wound healing assay illustrating increased migratory capacity of OVCAR3 cells upon *PABPC3* overexpression. **J** Quantification of wound healing assay results in OVCAR3 cells. **K** Real-time qPCR analysis of *CLDN1* and *CLDN9* expression levels. **L** In vivo bioluminescence imaging performed on days 12, 22, and 29 following intraperitoneal injection of control and *PABPC3*-overexpressing ID8 cells into nude mice. **M** Hematoxylin and eosin (HE) staining and CLDN1 immunohistochemical staining of liver tissue from injected mice. **N** Quantification of CLDN1 immunohistochemical staining results. **O** Proportion of mice with SKOV3 tumors in the mesentery. **P** In vivo bioluminescence imaging performed on day 23 following intraperitoneal injection of control and *PABPC3*-overexpressing SKOV3 cells into nude mice. **Q** Hematoxylin and eosin (HE) staining and CLDN1 immunohistochemical staining of mesentery tissue from injected mice. **R** Quantification of CLDN1 immunohistochemical staining results. Bar charts represent the mean ± SD. Statistical significance was determined using a two-sided unpaired Student’s *t*-test with a 95% confidence interval. Each experiment was independently repeated three times with consistent results. *p < 0.05, **p < 0.01, ****p < 0.0001. Scale bars, 300 µm (specific scale bars indicated in the figure).

To further examine the impact of *PABPC3* overexpression in ovarian cancer cells, we established a stable *PABPC3*-overexpressing cell line, referred to as Flag-*PABPC3*, in the ID8 cell line via lentiviral transduction. Western blot (WB) results confirmed stable overexpression of the *PABPC3* in ID8 cells by Flag tagging (Fig. 5A, unedited gel is added in Supplementary materials). Cell proliferation was then assessed using a CCK8 assay, which indicated that the proliferation rate of Flag-*PABPC3* cells was comparable to that of the control group (Fig. S2A). Similarly, colony formation assays revealed no significant differences in colony number or size between the Flag-*PABPC3* and control groups, suggesting that *PABPC3* overexpression does not notably affect cell proliferation (Fig. S2B, C). In contrast, Transwell migration assays demonstrated a marked increase in the migratory capacity of Flag-*PABPC3* cells compared to controls (Fig. 5B, C). Wound-healing assays further demonstrated that the migration rate of Flag-*PABPC3* cells was significantly higher than that of control cells (Fig. 5D, E). In order to further verify the effect of overexpression of *PABPC3*, we also constructed stable overexpression cell lines in human cell lines OVCAR3 and SKOV3 and performed the above experiments. The results showed that in OVCAR3 and SKOV3, overexpression of *PABPC3* did not change the proliferation rate and colony number of cells (Figs. 5F and S2D–J). Wound healing and Transwell assay also showed that the overexpression significantly increased the migration ability of the cells (Figs. 5G–J and S2K–N).

Additionally, RT-qPCR analysis revealed that the transcription levels of *Claudin* family members, *CLDN1* and *CLDN9*, were significantly elevated in the Flag-*PABPC3* group (Fig. 5K). These findings suggested that increased *PABPC3* expression promotes cell migration in ovarian cancer cells while exerting minimal impact on cell proliferation.

### *PABPC3* promoted cancer metastasis in vivo by reducing CLDN1 expression

Our in vitro findings indicated that *PABPC3* influences ovarian cancer cell metastasis through modulation of tight junctions. To verify the critical role of PABPC3 in vivo, we transfected both control and Flag-*PABPC3* groups with a luciferase reporter gene [28, 29]. Luciferase-labeled ID8 and Flag-*PABPC3* cell lines were then injected into nude mice to establish tumor metastasis models. Two injection methods were employed: intraperitoneal injection and tail vein injection, and luciferase activity in the mice was measured on days 12, 22, and 29 after injection.

The results showed that, following intraperitoneal injection of tumor cells, luciferase activity in the Flag-*PABPC3* group significantly increased over time, while the control group showed only a slow increase in fluorescence intensity. This finding suggests that ID8 cells with high *PABPC3* expression possess greater metastatic capacity in vivo (Figs. 5L and S3A). On day 29, we collected samples, counted the tumor-bearing organs, and performed H&E staining on liver tissues. The staining indicated that the Flag-*PABPC3* group had larger tumor areas. Immunohistochemical staining for CLDN1 in liver tissues further revealed significantly lower CLDN1 expression near tumors in the *PABPC3*-overexpressing group compared to the control group (Fig. 5M, N). Similarly, following tail vein injection of ovarian cancer cells, tumors were primarily localized to the lung region, and mice in the *PABPC3* overexpression group exhibited higher fluorescence intensity, indicating increased metastasis capacity in vivo (Fig. S3B, C). On day 29, TSA staining was performed to assess the co-expression of CLDN1 and Flag in lung tissues with results showing significantly reduced CLDN1 levels in the Flag-*PABPC3* group compared to controls (Fig. S3D, E).

To enhance the relevance of our findings and validate the robustness of our experimental model, we extended our studies from the murine ovarian cancer cell line ID8 to the human ovarian cancer cell line SKOV3. First, we injected luciferase-labeled SKOV3 and Flag-*PABPC3* cells into nude mice by intraperitoneal injection or tail vein injection to establish ovarian cancer metastasis models. After 23 days, fluorescence was photographed and luciferase activity was detected. After the tissue samples were collected, more tumors were found in the mesentery of the Flag-*PABPC3* group (Figs. 5O and S3F). Consistently, fluorescence imaging results showed that the Flag-PABPC3 group had a more scattered distribution in the intraperitoneal injection model (Fig. 5P). Through immunohistochemistry, it was also confirmed that in the tumors located on the mesentery, the *PABPC3* overexpression group had a lower level of CLDN1. (Fig. 5Q, R). Similarly, in tail vein injected mice, Flag-*PABPC3* group had higher fluorescence intensity, indicating increased metastatic capacity in vivo (Fig. S3G). Lung tissues were collected for TSA assay, and the results showed that CLDN1 levels were significantly reduced in the Flag-*PABPC3* group, consistent with the ID8 mouse model (Fig. S3H, I).

Taken together, these findings indicate that *PABPC3* overexpression enhances metastatic capacity in vivo, with reduced expression of Claudin family members CLDN1 suggesting decreased tight junction integrity, which may be a potential reason for tumor metastasis.

### High levels of PABPC3 enhanced the drug sensitivity and predict poor prognosis

**Fig. 6:**
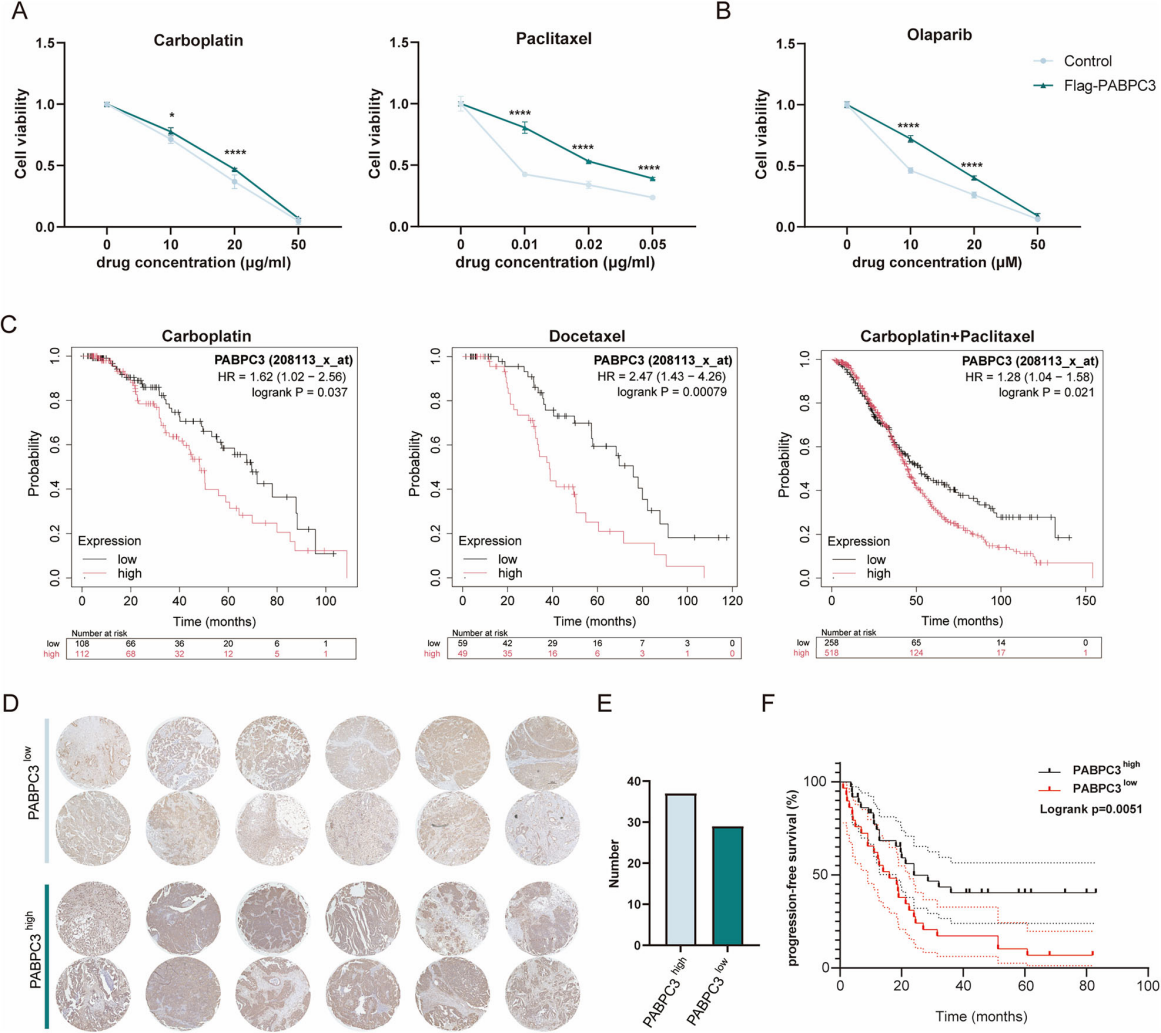
High PABPC3 expression diminishes drug sensitivity and predicts poor prognosis. **A** The CCK8 assay was performed to evaluate the proliferation activity of *PABPC3*-overexpressing ID8 cells following treatment with carboplatin (0, 10, 20, 50 µg/mL) and paclitaxel (0, 0.01, 0.02, 0.05 µg/mL) in a concentration-dependent manner (IC₅₀ of carboplatin: control = 15.38 µg/mL, Flag-PABPC3 = 18.07 µg/mL; IC₅₀ of paclitaxel: control = 0.0057 µg/mL, Flag-PABPC3 = 0.0296 µg/mL). **B** The CCK8 assay assessed the proliferation activity of *PABPC3*-overexpressing ID8 cells after treatment with Olaparib (0, 10, 20, 50 µM) under a concentration gradient (IC₅₀: control = 9.21 µM, Flag-PABPC3 = 16.24 µM). **C** Survival curves were generated for patients stratified into high and low *PABPC3* expression groups using database-derived data after chemotherapy. The expression threshold was set at the median *PABPC3* level, and data were obtained from GEPIA2. **D** Tissue microarray staining results were used to categorize samples into high and low *PABPC3* expression groups. Immunohistochemistry (IHC) scoring criteria were defined as follows: samples with strong cytoplasmic staining in more than 40% of tumor cells were classified as high expression, whereas those with staining in less than 40% were categorized as low expression. **E** Statistical analysis of *PABPC3* immunohistochemical staining results. **F** The Kaplan–Meier method was used to generate survival curves, comparing progression-free survival (PFS) between the high and low *PABPC3* expression groups. The omental metastasis tumor samples and paired progression-free survival information were collected from 66 patients who were diagnosed with ovarian cancer at the First Affiliated Hospital of Soochow University. Bar charts and line charts represent the mean ± SD. Statistical significance was determined using a two-sided unpaired Student’s *t*-test with a 95% confidence interval. Each experiment was independently repeated three times with consistent results. *p < 0.05, ****p < 0.0001. Scale bars, 300 µm.

In ovarian cancer treatment, most patients experience relapse due to drug susceptibility, often leading to fatal outcomes. To investigate the impact of *PABPC3* on drug sensitivity, we treated ID8 and Flag-*PABPC3* cell lines with the common first-line chemotherapeutic agents, including carboplatin [30] and paclitaxel [31, 32], as well as the PARP inhibitor Olaparib [33, 34], which has been increasingly used to treat ovarian cancer. Cell viability following drug treatment was assessed using the CCK8 assay. After being treated with varying drug doses for two days, Flag-*PABPC3* cells consistently showed higher viability across the paclitaxel, carboplatin, and PARP inhibitor Olaparib groups compared to controls, indicating that high *PABPC3* levels decrease drug sensitivity and reduce PARP inhibitor sensitivity (Fig. 6A, B). The same result was demonstrated by the clone formation assay (Fig. S4A). Furthermore, to validate our findings, we conducted experiments in three human cell lines (SKOV3, A2780, and OVCAR3) with PABPC3 overexpression and verified their sensitivity to paclitaxel (Fig. S4B–D). Radiation therapy is another common approach in treating ovarian cancer. To assess whether PABPC3 affects radiotherapy response, we exposed ovarian cancer cells to a range of radiation doses. The results showed no significant difference between the two groups following radiation treatment, suggesting that PABPC3 does not notably impact the radiotherapy resistance in ovarian cancer cells (Fig. S4E, F).

To further examine the role of PABPC3 in clinical outcomes, we analyzed the overall survival data based on *PABPC3* levels in patients receiving chemotherapy. We found that patients with high PABPC3 expression had significantly shorter overall survival, regardless of treatment with carboplatin, docetaxel, or a combination of carboplatin and paclitaxel (Fig. 6C). Additionally, to further validate our findings, we established a cohort of 66 patients with metastatic ovarian cancer, then we stratified samples by *PABPC3* expression (PABPC3^low^ and PABPC3^high^) and analyzed progression-free survival. The PABPC3^high^ group exhibited significantly shorter progression-free survival compared to the PABPC3^low^ group (Fig. 6D–F).

These results demonstrated that PABPC3 expression levels influence the drug sensitivity and progression-free survival in ovarian cancer, suggesting *PABPC3* as a potential therapeutic target or prognostic marker, offering new insights into the clinical management of ovarian cancer.

## Discussion

Ovarian cancer is a leading cause of cancer-related mortality among women, posing a serious threat to women’s health. The high mortality rate is often linked to metastasis, with most ovarian cancer patients ultimately succumbing to intraperitoneal tumor spread [35]. Unlike other malignancies, ovarian cancer primarily disseminates through the peritoneal cavity, characterized by superficial infiltration. It rapidly propagates within the abdominal cavity, with the omentum and peritoneum being the most common sites for distant metastasis [36]. To elucidate the mechanisms underlying ovarian cancer metastasis and improve prognostic predictions, we performed snRNA seq to analyze gene expression differences between primary and metastatic tumors. Considering the heterogeneity among individual samples, we combined all samples for an integrated analysis rather than analyzing each patient separately, in order to capture consistent metastatic signatures. Integrating these findings with public database survival analyses, we identified that *PABPC3* expression levels may correlate with tumor metastasis and patient prognosis. Notably, *PABPC3* expression was significantly elevated in metastatic lesions compared to primary tumors, and patients with high *PABPC3* levels demonstrated reduced overall survival. Thus, *PABPC3* may serve as a potential biomarker for predicting patient outcomes and metastasis. The PABP family comprises essential RNA-binding proteins that primarily bind to poly(A) tail at the 3′ end of mRNA, thereby regulating mRNA stability, translation, and transport [37]. Previous studies have shown that *PABPC1*, another member of the PABP family, plays a critical role in gastric cancer metastasis by promoting EMT through stabilizing PAK1 mRNA [38]. Additionally, in bladder cancer, PABPC1 enhances cell migration, invasion, and gemcitabine resistance through the PTK2-SETDB1 pathway [39]. While *PABPC3*’s specific effects on cell proliferation and metastasis in ovarian cancer patients remain unexplored.

In this study, we demonstrate that PABPC3 levels significantly influence the migratory and proliferative capacity of ovarian cancer cells. In vitro experiments revealed that silencing *PABPC3* markedly reduced their migration and proliferation, whereas overexpression of *PABPC3* enhanced their migratory potential. These findings indicated that high PABPC3 expression substantially increases the metastatic capacity of ovarian cancer cells. Furthermore, in vivo experiments indicated that PABPC3-overexpressing ovarian cancer cells accelerated metastasis activity in mice. We acknowledge that our findings would be more robust if further validated in human HG-SOC models, such as patient-derived xenografts or patient-derived organoids. It would be valuable to reassess our findings in these models before advancing to clinical investigations. Collectively, these results further suggested the essential role of PABPC3 in tumor metastasis.

Previous studies have highlighted alterations in tight junction protein expression across various human malignancies. Tight junctions are essential for maintaining cell-cell adhesion, regulating cellular permeability, and signaling [40, 41]. During tumor progression, cancer cells frequently downregulate the expression of tight junction proteins, thereby disrupting intercellular junctions and facilitating invasion and metastasis [42]. CLDN1, a vital component of tight junction complexes, functions as an inhibitor of cancer invasion by preserving epithelial barrier integrity and regulating cell permeability. Reduced expression of CLDN1 is closely associated with tumor metastasis. Studies indicate that CLDN1 overexpression may enhance tumor cell proliferation and migration [41], while its downregulation can compromise tight junction integrity and augment cancer cell invasiveness [43, 44]. Notably, alterations in CLDN1 expression have been identified as important biomarkers of tumor progression in various cancers, including breast, ovarian, and liver cancer [45]. In our study, we confirmed that high PABPC3 expression correlates with lower CLDN1 levels through qPCR, IHC, and TSA assays. Conversely, silencing *PABPC3* in cells resulted in increased CLDN1 levels, further suggesting that PABPC3 promotes ovarian cancer metastasis.

Standard treatment for ovarian cancer typically involves surgical intervention followed by platinum/paclitaxel-based chemotherapy [2, 46, 47], which effectively eliminates proliferative tumor cells. However, most patients eventually experience relapse due to drug-resistant ovarian cancer, a primary cause of mortality [48, 49]. In this study, we also investigated the relationship between *PABPC3* levels and chemotherapy resistance and PARP inhibitors, which have been commonly used to treat ovarian cancer in recent years. ID8 cells were treated with carboplatin, paclitaxel, and the PARP inhibitor Olaparib [33, 47, 50]. Our results demonstrated that ovarian cancer cells with elevated *PABPC3* expression exhibited decreased drug sensitivity to chemotherapeutic agents and decreased sensitivity to PARP inhibitors. It suggests that *PABPC3* may contribute to tumor resistance and could serve as a potential therapeutic target. Additionally, survival analyses utilizing public databases revealed that patients with high *PABPC3* expression experienced lower survival rates than those with low expression following chemotherapy. This observation was corroborated by clinical sample survival analyses. Follow-up studies of ovarian cancer patients indicated that those with high *PABPC3* expression had a higher mortality rate post-treatment compared to patients with low expression, further supporting the role of *PABPC3* in drug sensitivity. Given that radiotherapy is another effective cancer treatment, we further tested whether PABPC3 overexpression confers radioresistance in ovarian cancer. Our experiments revealed that PABPC3 overexpression does not alter the sensitivity of ovarian cancer cells to radiation (Fig. S4E, F). Based on these findings, patients with high PABPC3 expression may derive greater therapeutic benefits from radiotherapy as an alternative to chemotherapy.

In conclusion, we found that *PABPC3* expression levels are significantly elevated in metastatic lesions from the same patient. Through in vitro and in vivo experiments, we confirmed that high *PABPC3* expression enhances the migratory capacity of ovarian cancer cells, indicating that *PABPC3* plays a crucial role in ovarian cancer metastasis. While its high expression may facilitate metastasis, the precise underlying mechanisms warrant further investigation. Our findings suggest that *PABPC3* expression levels in tumor cells may influence the tumor microenvironment during ovarian cancer metastasis, thereby promoting metastatic spread. Therefore, *PABPC3* represents a promising therapeutic target for ovarian cancer treatment.

## Supplementary information

Figure S1

Figure S2

Figure S3

Figure S4

Figure S5

Supplementary table 1

Supplementary table 2

SUPPLEMENTAL MATERIAL

uncropped original western blots

## Data Availability

All images and raw data for statistical analysis in this study are available from the corresponding author upon reasonable request. Single-nucleus RNA sequencing data (HRA009020) was deposited on GSA for Human (https://ngdc.cncb.ac.cn/gsa-human/).
