# Supplementary figures and images for: PABPC3 drives ovarian cancer metastasis and drug sensitivity by downregulating CLDN1 expression
Source: Cell Death Dis. 2025 Nov 17;16(1):840. doi: 10.1038/s41419-025-08151-5 (PMC12624041; doi:10.1038/s41419-025-08151-5)

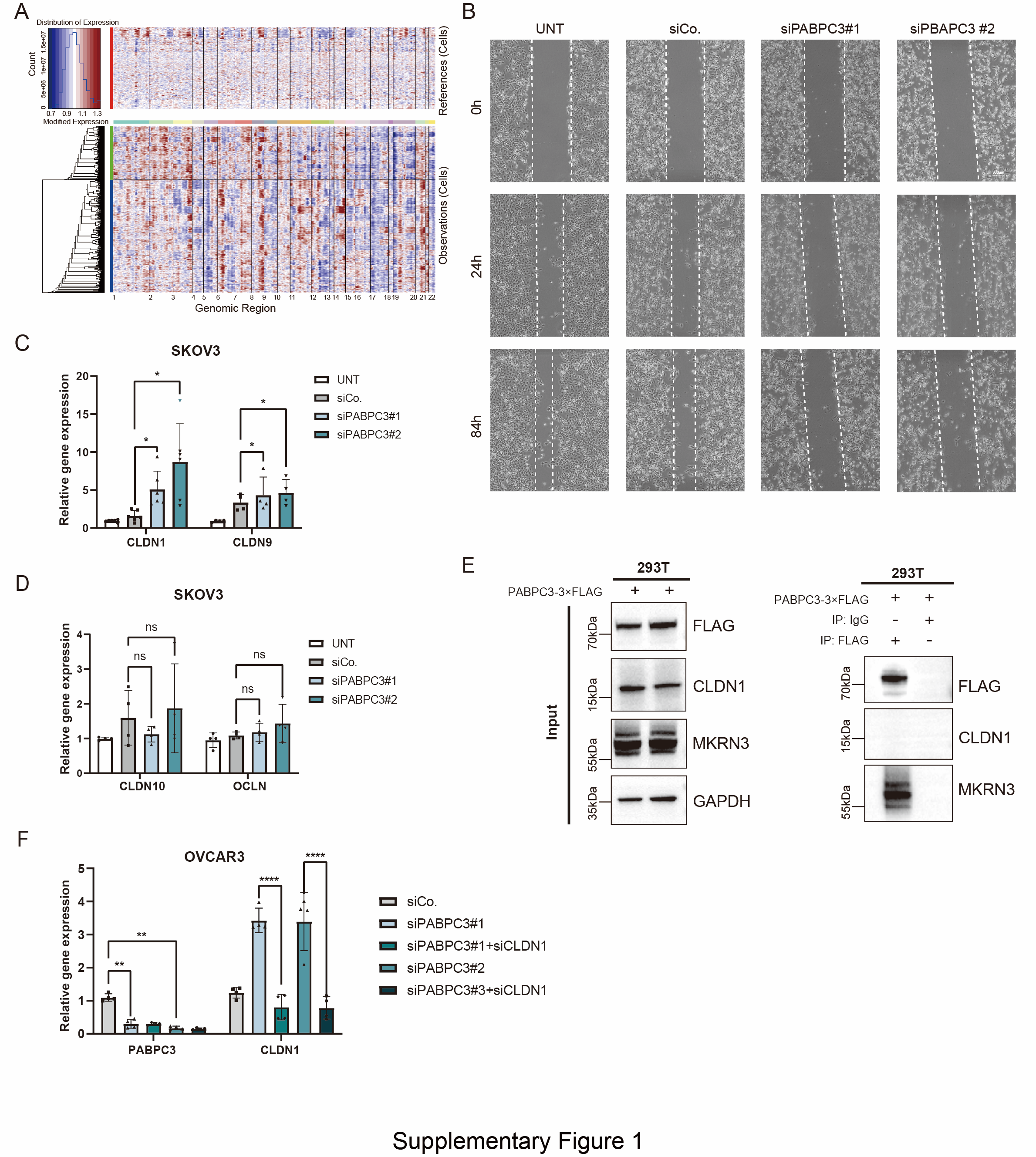

Supplement: Supplementary file 1 — Figure S1 [file 41419_2025_8151_MOESM1_ESM.tif]

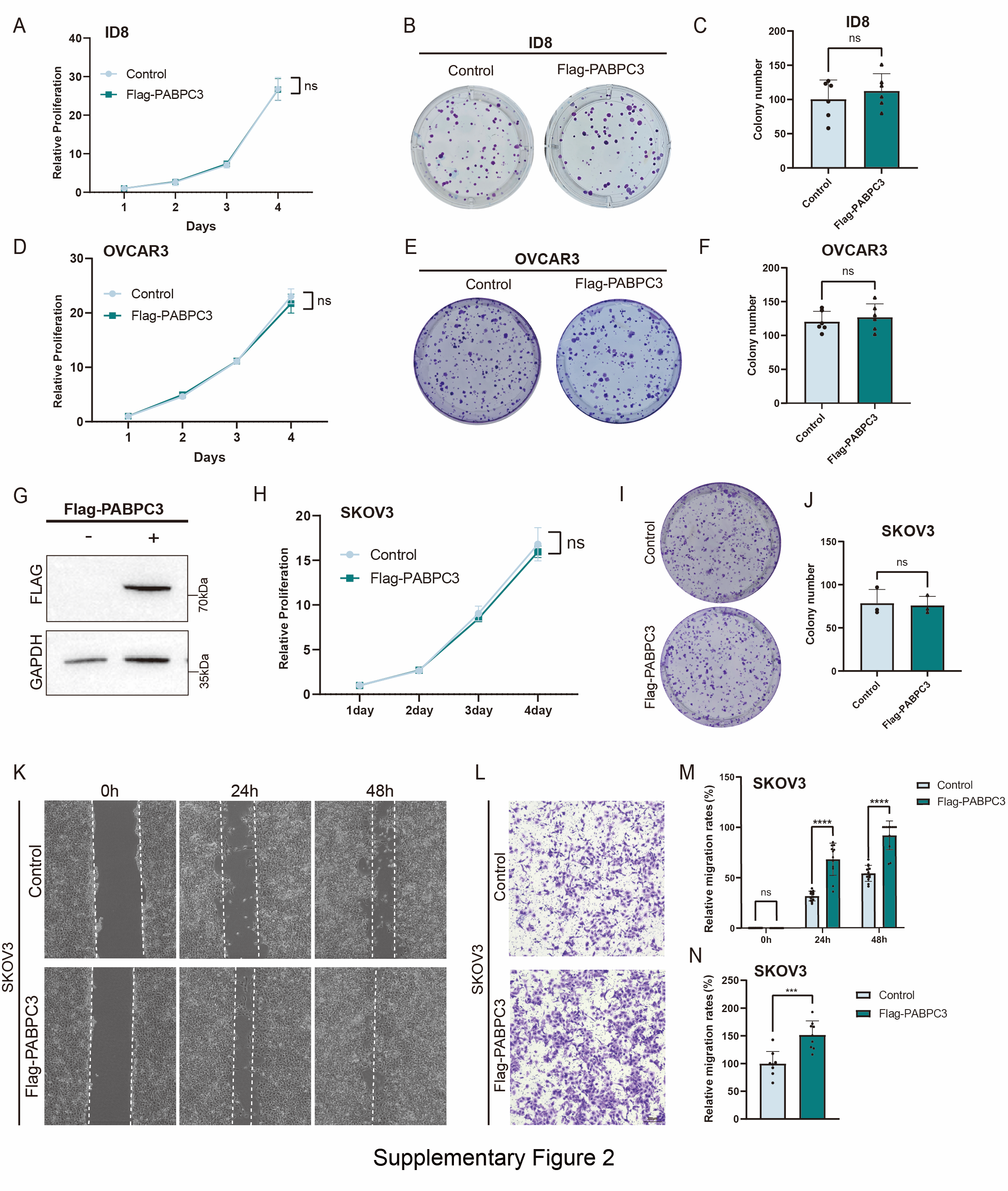

Supplement: Supplementary file 2 — Figure S2 [file 41419_2025_8151_MOESM2_ESM.tif]

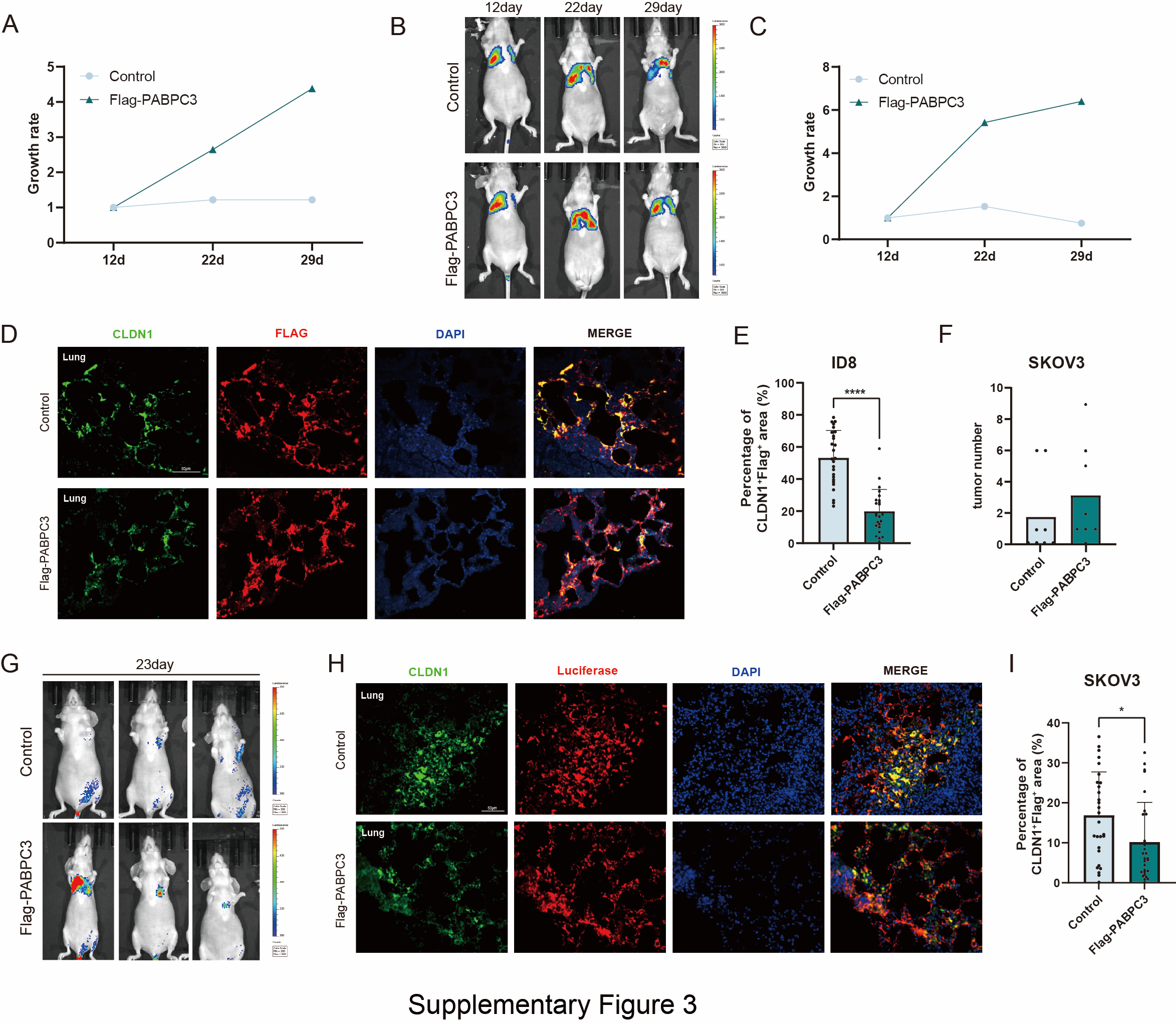

Supplement: Supplementary file 3 — Figure S3 [file 41419_2025_8151_MOESM3_ESM.tif]

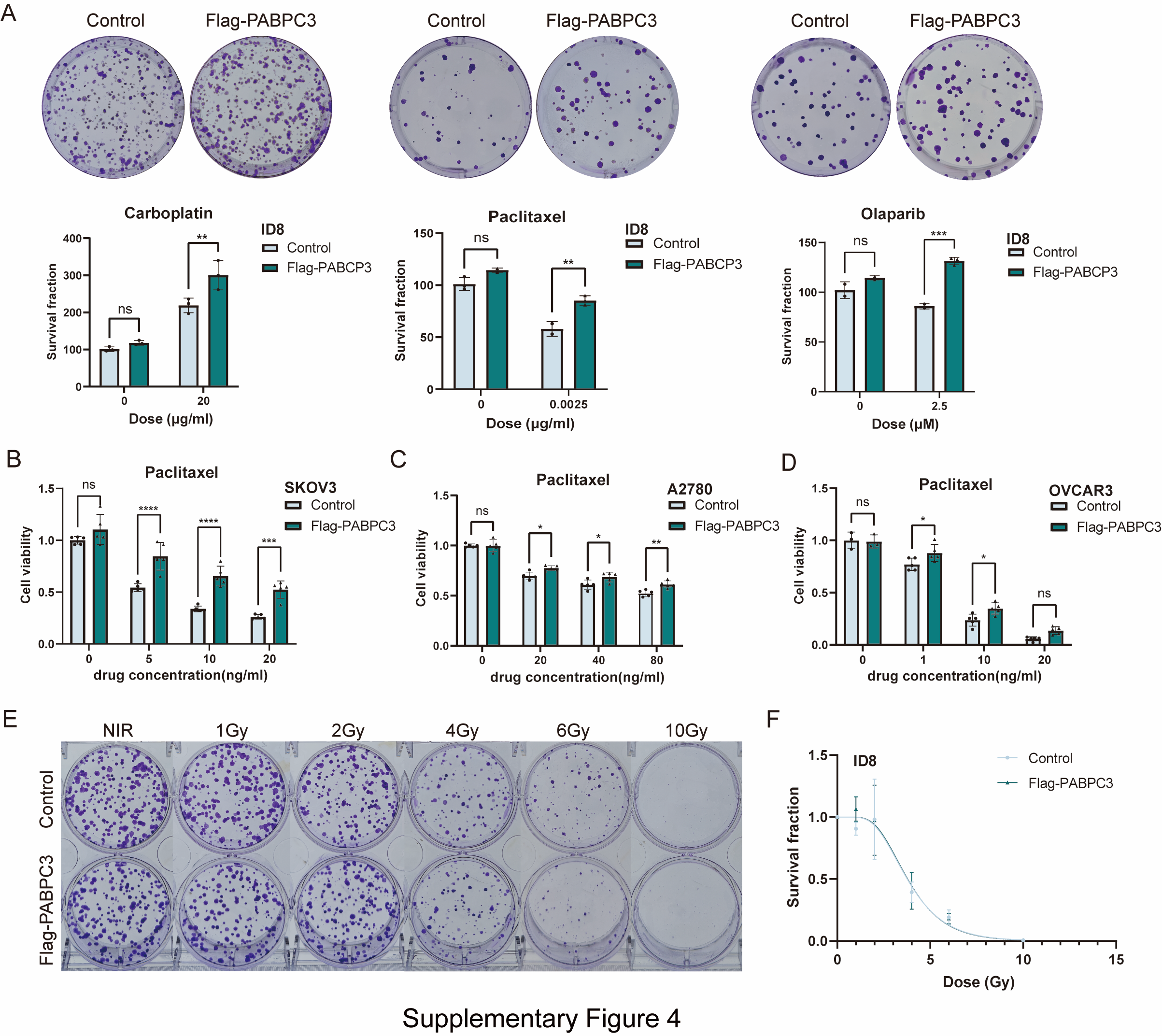

Supplement: Supplementary file 4 — Figure S4 [file 41419_2025_8151_MOESM4_ESM.tif]

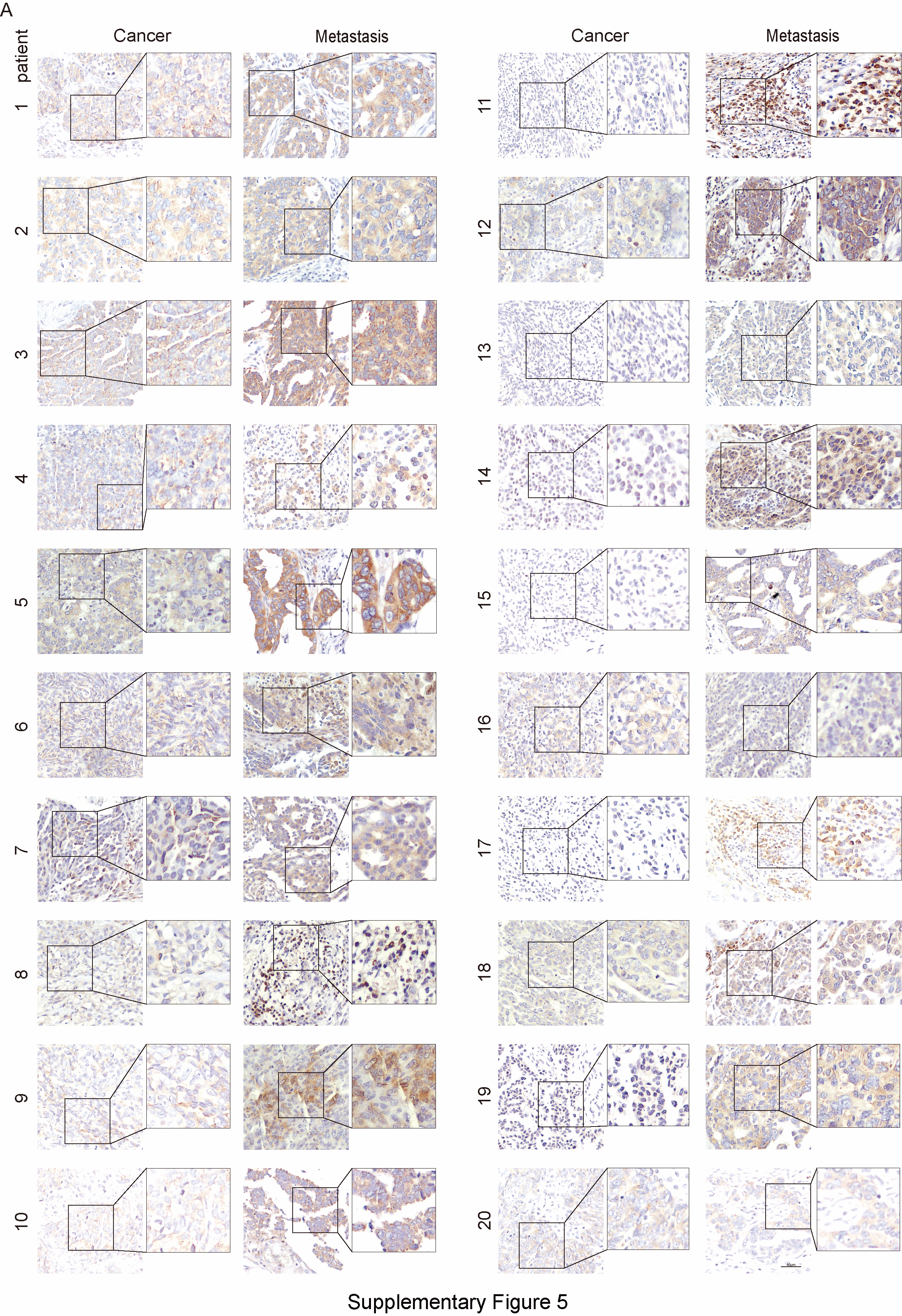

Supplement: Supplementary file 5 — Figure S5 [file 41419_2025_8151_MOESM5_ESM.tif]
