# Supplementary material for: PABPC3 drives ovarian cancer metastasis and drug sensitivity by downregulating CLDN1 expression
Source: Cell Death Dis. 2025 Nov 17;16(1):840. doi: 10.1038/s41419-025-08151-5 (PMC12624041; doi:10.1038/s41419-025-08151-5)
Supplement: Supplementary file 8 — SUPPLEMENTAL MATERIAL [file 41419_2025_8151_MOESM8_ESM.pdf]

Supplementary materials for PABPC3 Drives Ovarian Cancer Metastasis and Drug Sensitivity by Down-Regulating CLDN1 Expression

Hong Zhang[1]#, Yiping Lin[1]#, Mintao Ji[2]#, Yuhan Guo[1], Haisheng Liang[1], Kai Kang[1], Shuangshuang Lu[1], Zhisen Zhang[1], Yinyin Shu[2], Xiaoni Jin[1], Wenjuan Gan[3], Qian Xu[1], Youguo Chen[1]*, Yuhong Wang[1,4], Zhe Lei[1,4], Lingchuan Guo[1,4]*, Chunlin Shao[2]*, Lei Chang[1, 2]*

Animal models

The mice were purchased from the Shanghai Model Organisms Center, Inc., and all animal experiments were conducted in accordance with the requirements of the Animal Care and Use Committee of Soochow University. All studies involving mice were approved by the Soochow University Institutional Animal Care and Use Committee. For the 6-8-week-old immunodeficient nude mice, ID8 cells transfected with a luciferase reporter gene and ID8 cells overexpressing PABPC3 were injected via intraperitoneal or tail vein injection, with 5 × 10^6 cells injected per mouse. The mice were randomly divided into groups (n=6 per group). On days 12, 22, and 29 post-injection, luciferin (Meilunbio, MB1834-2) was injected, and the mice were imaged using the IVIS imaging system (PerkinElmer, IVIS Lumina XR III). On day 29, the mice were sacrificed, and tissues such as liver and lungs were collected. The SKOV3 constructed nude mouse model was similar to ID8 and imaged at 23 days. The tissues were fixed in formalin and embedded in paraffin for histological analysis. The animal experiment adopted a blinded design. Throughout the experiment, operators were unaware of the animals' group assignments.

RNA interference

In this study, siRNA was used to influence gene expression in SKOV3 cells and OVCAR-3 cells. The siRNA was initially transported in a lyophilized powder form and was immediately centrifuged upon receipt, then dissolved in sterilized nuclease-free water. The cells were seeded into six-well plates at a density of 30-40% to reach 50% density by the next day for transfection. They were divided into four groups: control group (no treatment), siCo. (transfected with siALLstar, QIAGEN, SI03650318), siPABPC3#1 group (sequence: GCGTATGTGAACTTCCAGCAT), and siPABPC3#2 group (sequence: GCCACTAAAGCAGTTACAGAA). In addition, siCLDN1 was used

in the rescue experiment (sequence: CTGGGAGTGATAGCAATCTTT). For the siRNA transfection, 0.8 μL of the siRNA solution was mixed with opti-MEM, followed by the addition of 4 μL of transfection reagent to ensure thorough mixing of the mixture. After gently pipetting and mixing, the solution was incubated at room temperature for 15 minutes to form the transfection complex. The transfection complex was added to a medium lacking dual antibiotics and incubated for 24 hours. After 24 hours, the medium was replaced with complete culture medium, and the cells were cultured for an additional 48 hours before using quantitative real-time PCR to verify transfection efficiency.

Luciferase reporter assay

The cells were seeded in twelve-well plates and luciferase assays were performed using the pGL4.1-CLDN1 promoter cotransfected with the CMV-β-gal plasmid. Before transfection, cells were pretreated with the corresponding siRNA, and luciferase activity was detected by firefly luciferase reporter gene detection kit (Beyotime, China) and β-galactosidase detection kit (Beyotime, China) 48h after transfection. The luciferase reporter plasmid for CLDN1 was purchased from Addgene (#46387).

Lentiviral packaging and overexpression

A three-plasmid system was first used to package lentivirus in HEK293T cells. This system includes a transfer plasmid containing the target gene [pLV3-CMV-PABPC3(human)-3×FLAG-Puro], a packaging plasmid (psPAX2), and an envelope plasmid (PMD2VSV4). These three plasmids were co-transfected into HEK293T cells, and after 48-72 hours of culture, the cell culture supernatant was collected, which contained the packaged lentiviral particles. Subsequently, a filter (SORFA, 0.22 μm) was used to directly filter out cell debris, and the supernatant was collected in centrifuge tubes and stored at -20 °C.

Cells in good condition and the logarithmic growth phase were used for transfection. After digestion and centrifugation, the resuspended cells were evenly plated onto culture dishes and incubated in a cell culture incubator. When the cell density reached 30-40%, lentiviral transduction was performed. After removing the old culture medium, 4 ml of fresh medium and 4 ml of viral solution were added and mixed, and the dishes were placed in the cell culture incubator for further incubation. After 48 hours, the culture medium was replaced, and puromycin treatment was added. A blank control group was established, and an equal amount of puromycin treatment was added to the control group. A stable PABPC3-overexpressing cell line was obtained when all cells in the control group died. The transduction efficiency was confirmed by Western blot

analysis.

Fluorescein reporter transfection

An expression vector containing the luciferase gene was designed, and the constructed vector was introduced into target cells via lentiviral transduction. To screen for stably expressing clones, cells were cultured under puromycin selection pressure, with a blank control group treated with an equal amount of puromycin. A stable expression cell line was obtained when all cells in the control group had died. After selection, the transfection was assessed using a luciferase substrate (Meilunbio, MB1834-2). Luciferase catalyzed light emission, and the luminescence intensity was quantitatively measured with a microplate reader.

CCK8 assay

The CCK8 assay was used to assess cell viability. For SKOV3 cells and OVCAR-3 cells transfected with siPABPC3, 24 hours post-transfection, the cells were digested and centrifuged, then seeded into 96-well plates at a density of 3,000 cells per well, with five replicates per group. The 96-well plates were incubated at intervals of 0, 24, 48, and 72 hours in the cell incubator. At each designated time point, one 96-well plate was removed from the incubator, the medium was discarded, and 100 μl of culture medium containing 10% CCK8 reagent (New Cell & Molecular, C6005) was added to each well. The plate was returned to the incubator for 1 hour, and absorbance was measured at 450 nm using a microplate reader. For SKOV3, OVCAR3, and ID8 cell lines overexpressing PABPC3, cells in logarithmic growth phase were taken from the control and Flag-PABPC3 groups, logarithmic-phase cells from both groups were digested and seeded into 96-well plates at a density of 2,000 cells per well, with five replicates per group. Cells were incubated at intervals of 0, 24, 48, and 72 hours, with medium replaced by CCK8-containing medium at each time point. Cells were incubated at 37°C for 30 minutes, and absorbance was measured at 450 nm using a microplate reader. For drug treatment in the ID8 cell line, logarithmic-phase ID8 cells and Flag-PABPC3 cells were digested and seeded into 96-well plates at a density of 3,000 cells per well. After 24 hours of incubation, the medium was replaced with drug-containing medium: carboplatin (concentration gradient of 0, 10, 20, 50 μg/ml), paclitaxel (concentration gradient of 0, 0.01, 0.02, 0.05 μg/ml), or Olaparib (concentration gradient of 0, 10, 20, 50 μM). Similarly, SKOV3, A2780, and OVCAR3 cells with PABPC3 overexpression and control cells were treated with paclitaxel at different concentrations (SKOV3: 0, 5, 10, 20 ng/mL; A2780: 0, 20, 40, 80 ng/mL; OVCAR3: 0, 1, 10, 20 ng/mL). CCK8 assays were performed 48 hours after treatment as described below. After 48 hours of drug treatment, the medium was discarded, and 100 μl of medium containing 10%

CCK8 reagent was added to each well. The plate was incubated in the cell incubator for 30 minutes, and absorbance at 450 nm was measured using a microplate reader.

Colony formation assay

The colony formation assay was used to assess cell survival. For SKOV3 cells and OVCAR-3 cells, after transfection with siPABPC3 and 24 hours of culture, the cells were digested and centrifuged, and then seeded into six-well plates at a density of 5,000 cells per well, with three replicates. The six-well plates were gently shaken to ensure even distribution of the cells and then placed in the incubator for one weeks of culture. For the colony formation assay, logarithmic-phase ID8 and PABPC3-overexpressing ID8 cells were digested, centrifuged, and seeded into six-well plates at a density of 150 cells per well in triplicate. Colonies were allowed to form for 7 days before sample collection. Similarly, SKOV3 and PABPC3-overexpressing SKOV3 cells were seeded at 1,000 cells per well, and OVCAR3 and PABPC3-overexpressing OVCAR3 cells were seeded at 500 cells per well, both in triplicate. Colonies from SKOV3 and OVCAR3 cells were collected after 14 days. For ID8 cells treated with radiation gradients, cells received doses of 0 Gy, 1 Gy, 2 Gy, 4 Gy, 6 Gy, and 10 Gy, respectively, and were seeded into six-well plates at a density of 300 cells per well, with three replicates. After 7 days of incubation, samples were collected. For drug-treated ID8 cells, 150, 300, 600, and 1200 cells per well were seeded into six-well plates, and treated with gradient concentrations of drugs: carboplatin (2.5, 5, 10, 20 μg/ml), paclitaxel (0.0025, 0.005, 0.01, 0.02 μg/ml), and Olaparib (2.5, 5, 10, 20 μM). After 24 hours of drug treatment, the medium was replaced with drug-free complete medium. Samples from ID8 cells and PABPC3-overexpressing ID8 cells were collected on day 7 after seeding. The collected colony plates were first rinsed by removing the medium and washing three times with PBS. Cells were then fixed with 70% ethanol for 15 minutes, after which ethanol was removed, and the colonies were stained with crystal violet for 10 minutes. The plates were gently rinsed under a steady stream of running water and air-dried at room temperature. Colonies were made visible for observation and counting under the plate.

Transwell assay

For the Transwell assay with SKOV3 cells and OVCAR-3 cells, after transfection with siPABPC3, the cells were cultured for 24 hours, then digested and centrifuged. The cells were resuspended in serum-free medium, and 10,000 cells were seeded into the upper chamber of a 24-well plate (Corning, 3422). The lower chamber was filled with 600 μL of medium containing 10% FBS, and the plate was incubated for 42 hours in an incubator. For the overexpression experiments, ID8, OVCAR3, and SKOV3 cells in the

logarithmic growth phase were taken, digested, centrifuged, and then subjected to migration assays. The cells were resuspended in serum-free medium, and 10,000 cells were seeded into the upper chamber of a 24-well plate. The lower chamber was filled with 600 μL of medium containing 10% FBS, and incubated with ID8 for 30h and SKOV3 and OVCAR3 for 42h. After incubation, the cells were washed three times with PBS, fixed in 75% ethanol for 10 minutes, and stained with crystal violet for 5 minutes. Unmigrated cells were removed using a wet cotton swab. Five random fields were selected under the microscope, and the number of migrated cells was counted.

Wound healing assay

The wound healing assay was used to assess cell migration levels. For SKOV3 cells and OVCAR-3 cells, after 24 hours of siRNA transfection, the cells were resuspended and seeded into 24-well plates, with 1.5×10^5 cells per well. After 24 hours of incubation, a vertical scratch was made in the well using a pipette tip. The cells were washed three times with PBS to remove the detached cells, followed by the addition of serum-free medium. Images of the wound area were taken under a microscope at 0, 24, and 48 hours at the same observation point. For the ID8 cell line, logarithmic-phase cells were selected, resuspended, and seeded into 6-well plates with 1×10^6 cells per well. After 24 hours of incubation, a vertical scratch was made with a pipette tip, and the cells were washed three times with PBS to remove the detached cells. Serum-free medium was added, and images of the wound area were taken at 0, 4.5, 9, and 13.5 hours using a microscope, selecting 10 observation points per well. Similarly, SKOV3 and PABPC3-overexpressing SKOV3 cells were subjected to the same procedure, with images taken at 0, 24, and 48 hours; for OVCAR3 and PABPC3-overexpressing OVCAR3 cells, wound healing was monitored at 0, 12, 24, and 36 hours.

RT-qPCR

Logarithmic-phase cells were collected, and total RNA was extracted using the SimplyP Total RNA Extraction Kit (Bioer, BSC52). The quantity and quality of RNA were assessed using a Nanodrop 2000 spectrophotometer (Thermo), with most samples showing a 260/280 ratio greater than 1.8. Complementary DNA (cDNA) was synthesized using the HiScript III qRT SuperMix for qPCR (Vazyme, R223-01), and qPCR was performed using the ChamQ SYBR qPCR Master Mix (Vazyme, Q311-02) on a real-time quantitative PCR instrument (ThermoFisher Scientific). The forward and reverse primer sequences for the target genes are provided in supplementary table 2.

Tyramide signal amplification

After the paraffin-embedded tissue sections are dewaxed and rehydrated, antigen retrieval is performed, followed by blocking of endogenous peroxidase activity and non-specific binding sites. Different primary antibodies are applied in each round, and the samples are incubated overnight at 4°C. The antibodies were as follows: anti-CLDN1 (Zenbio, 680135), Flag (Sigma Aldrich, F1804-1MG), anti-Luciferase (ZenBio, R380996). Afterward, they are incubated with secondary antibodies (AiFang biological, AFIHC024) for 1 hour, followed by signal detection using fluorescent chromogens. Previous antibody complexes are removed by heating or chemical treatment, allowing for multiple rounds of labeling. The final staining results are obtained using a confocal microscope.

Figure Legends

Supplementary Figure 1. *PABPC3* knockdown significantly inhibited migration abilities while increasing CLDN1 levels.

A. The inferCNV method was used to predict copy number variations in epithelial cells from ovarian cancer samples, enabling the distinction of tumor cells. **B.** The wound healing assay indicated impaired migration in SKOV3 cells following *PABPC3* knockdown, with a reduction in wound closure capability. **C.** Real-time qPCR analysis of *CLDN1* and *CLDN9* expression levels. **D.** Real-time qPCR analysis of *CLDN10* and *OCLN* expression levels. **E.** Co-IP was conducted in 293T cells. The cells were co-transfected with Flag-PABPC3 and HA-MKRN3 for 48 hours. The cells were collected for immunoprecipitation against FLAG. **F**. Real-time qPCR analysis of *PABPC3* and *CLDN1* expression levels following transfection with siAllstar, siPABPC3, and siCLDN1. Bar charts and line charts represent the mean ± SD. Statistical significance was determined using a two-sided unpaired Student's *t*-test with a 95% confidence interval. Each experiment was independently repeated three times with consistent results. *p < 0.05, **p < 0.01, ****p < 0.0001. Scale bars, 300 μm.

Supplementary Figure 2. Results in vitro after *PABPC3*-overexpression treated.

A. The CCK8 assay was performed to assess the proliferation activity of ID8 cells following *PABPC3* overexpression. **B-C.** The colony formation assay demonstrated the clonogenic survival of ID8 cells after *PABPC3* overexpression. **D.** The CCK8 assay was performed to assess the proliferation activity of OVCAR3 cells following *PABPC3* overexpression. **E-F.** The colony formation assay demonstrated the clonogenic survival of OVCAR3 cells after *PABPC3* overexpression. **G**. An SKOV3 cell line with stable *PABPC3* overexpression was established, and its expression levels were validated by Western blot. **H.** The CCK8 assay was performed to assess the proliferation activity of

SKOV3 cells following *PABPC3* overexpression. **I-J.** The colony formation assay demonstrated the clonogenic survival of SKOV3 cells after *PABPC3* overexpression. **K**. Wound healing assay illustrating increased migratory capacity of SKOV3 cells upon *PABPC3* overexpression. **L**. Transwell assay demonstrating significantly enhanced migration of SKOV3 cells following *PABPC3* overexpression. **M.** Quantification of wound healing assay results in SKOV3 cells. **N**. Quantification of SKOV3 cell migration percentage in the Transwell assay after *PABPC3* overexpression. Bar charts and line charts represent the mean ± SD. Statistical significance was determined using a two-sided unpaired Student's *t*-test with a 95% confidence interval. Each experiment was independently repeated three times with consistent results. ***p < 0.001, ****p < 0.0001. Scale bars, 300 μm.

Supplementary Figure 3. *PABPC3* overexpression promotes metastasis and decreases CLDN1 levels.

A. Tumor cell proliferation was quantified by measuring bioluminescence intensity on days 12, 22, and 29. **B**. In vivo bioluminescence imaging conducted on days 12, 22, and 29 following tail vein injection of control and PABPC3-overexpressing ID8 cells into nude mice. **C.** Tumor cell proliferation was quantified by measuring bioluminescence intensity on days 12, 22, and 29. **D**. Fluorescent multiplex immunohistochemistry examining the co-localization of CLDN1 and Flag in lung tissue. **E**. Statistical analysis of CLDN1 and Flag co-localization across different regions. **F**. Statistics of the number of tumors per mouse. **G**. In vivo bioluminescence imaging conducted on day 23 following tail vein injection of control and PABPC3-overexpressing SKOV3 cells into nude mice. **H**. Fluorescent multiplex immunohistochemistry examining the co-localization of CLDN1 and Luciferase in lung tissue. **I**. Statistical analysis of CLDN1 and Luciferase co-localization across different regions. Bar charts and line charts represent the mean ± SD. Statistical significance was determined using a two-sided unpaired Student's *t*-test with a 95% confidence interval. Each experiment was independently repeated three times with consistent results. *p < 0.05, ****p < 0.0001. Scale bars, 300 μm.

Supplementary Figure 4. *PABPC3* overexpression decreases drug sensitivity.

A. The colony formation assay showed that *PABPC3*-overexpressing ID8 cell clones exhibited enhanced survival following treatment with carboplatin, paclitaxel, and Olaparib. **B**. The CCK8 assay was performed to evaluate the proliferation activity of *PABPC3*-overexpressing SKOV3 cells following treatment with paclitaxel (0, 5, 10, 20 ng/mL) (IC_{50}: control = 5.63 ng/mL, Flag-PABPC3 = 20.77 ng/mL). **C**. The CCK8

assay was performed to evaluate the proliferation activity of *PABPC3*-overexpressing A2780 cells following treatment with paclitaxel (0, 20, 40, 80 ng/mL) (IC_{50}: control = 661.8 ng/mL, Flag-PABPC3 = 720.3 ng/mL). **D**. The CCK8 assay was performed to evaluate the proliferation activity of *PABPC3*-overexpressing OVCAR3 cells following treatment with paclitaxel (0, 1, 10, 20 ng/mL) (IC_{50}: control = 2.997 ng/mL, Flag-PABPC3 = 5.506 ng/mL). **E**. The colony formation assay demonstrated the clonogenic survival of PABPC3-overexpressing ID8 cells following exposure to increasing doses of radiation (0, 1, 2, 4, 6, 10 Gy). **F**. Radiation dose-cell survival curve for PABPC3-overexpressing ID8 cells. Bar charts represent the mean ± SD. Statistical significance was determined using a two-sided unpaired Student's *t*-test with a 95% confidence interval. Each experiment was conducted independently three times with consistent results. *p < 0.05, **p < 0.01, ***p < 0.001, ****p < 0.0001.

Supplementary Figure 5. PABPC3 protein levels in clinical samples were stained by immunohistochemistry.

A. Immunohistochemical staining revealed *PABPC3* expression in primary and metastatic ovarian cancer tissues. The left and right panels represent primary and metastatic tissues, respectively, from the same patient. A total of 20 ovarian cancer clinical samples were analyzed.

Table Legends

Supplementary table 1. Clinical Characteristics of Patients

The table provides pathology ID, pathologic diagnosis, and progression-free survival (PFS, in months) of the enrolled patients

Supplementary table 2. Primers used for qRT-PCR.
