# Supplementary material for: PABPC3 drives ovarian cancer metastasis and drug sensitivity by downregulating CLDN1 expression
Source: Cell Death Dis. 2025 Nov 17;16(1):840. doi: 10.1038/s41419-025-08151-5 (PMC12624041; doi:10.1038/s41419-025-08151-5)
Supplement: Supplementary file 9 — uncropped original western blots [file 41419_2025_8151_MOESM9_ESM.pdf]

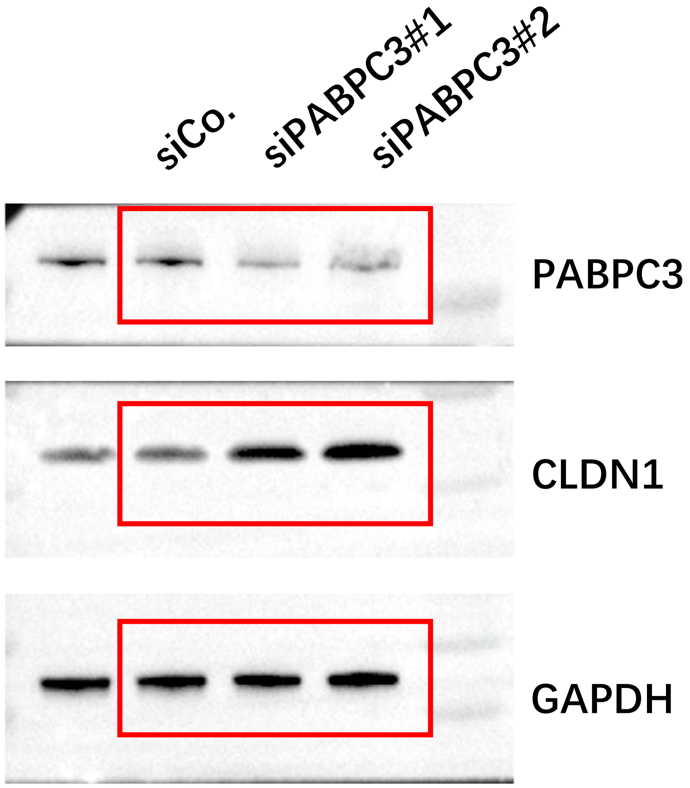

Full unedited gel for Figure 4F

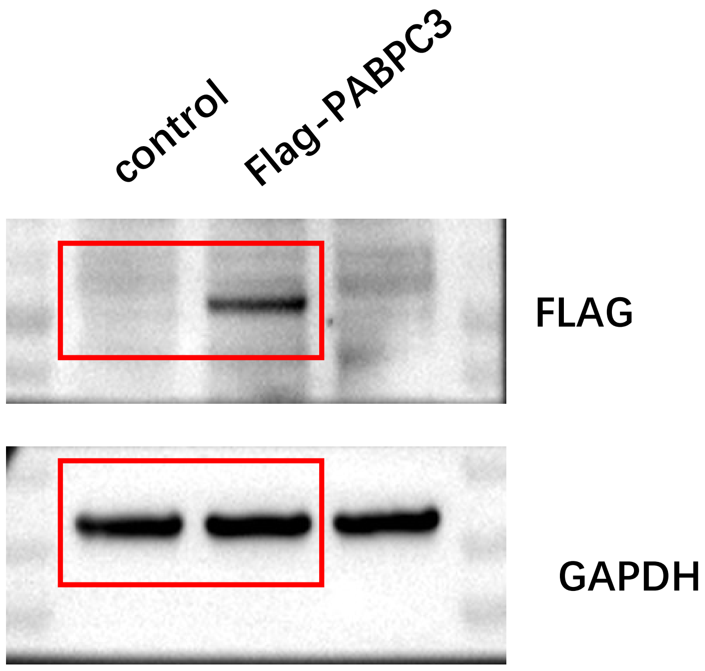

Full unedited gel for Figure 5A

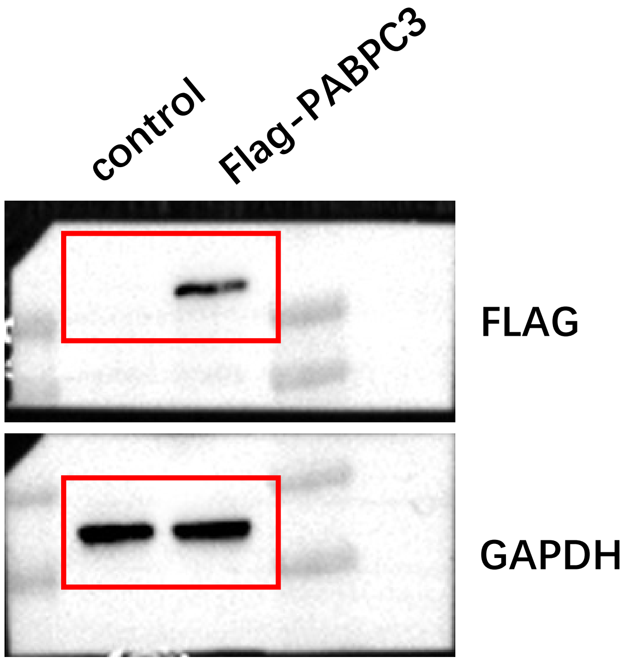

Full unedited gel for Figure 5F

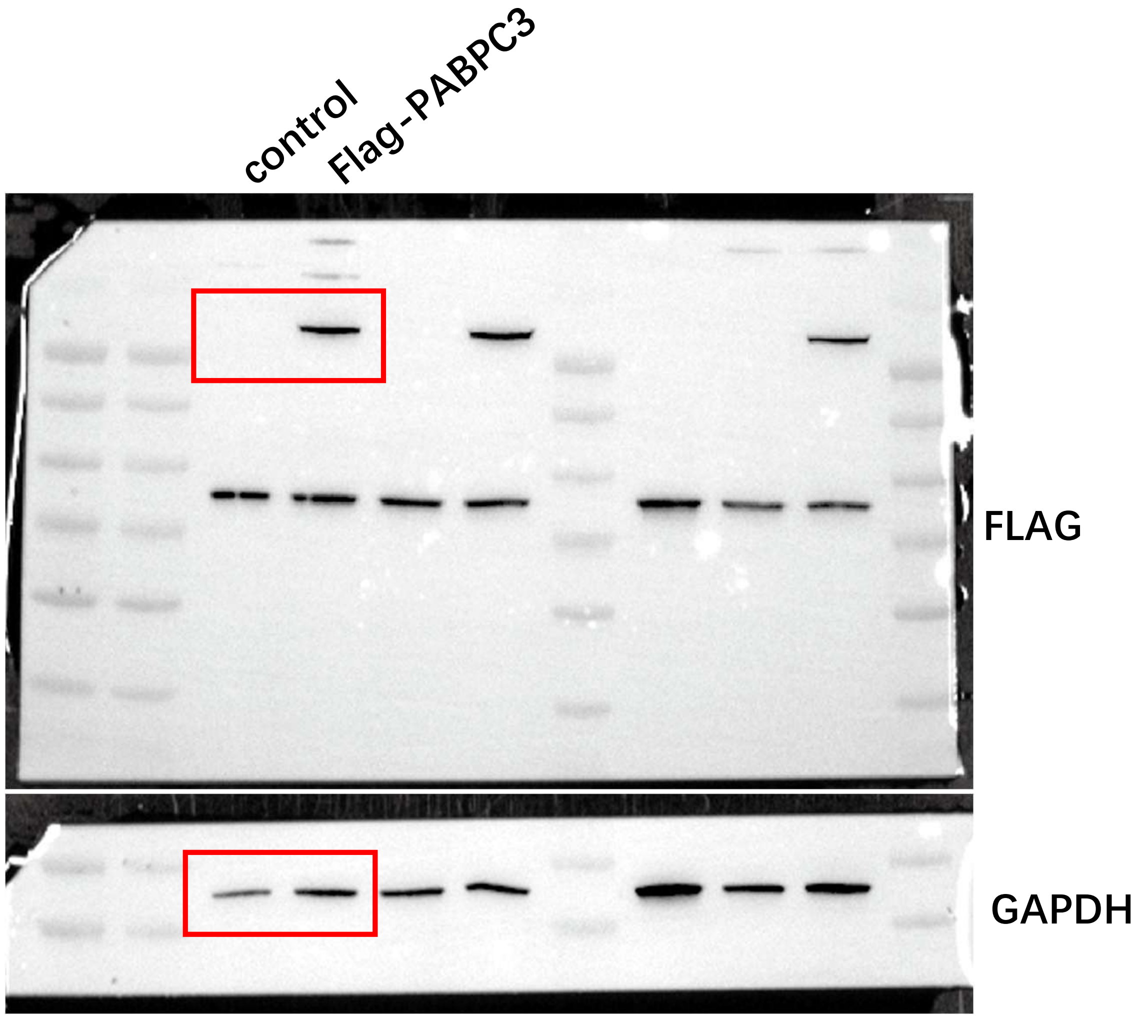

Full unedited gel for Supplementary Figure 2G

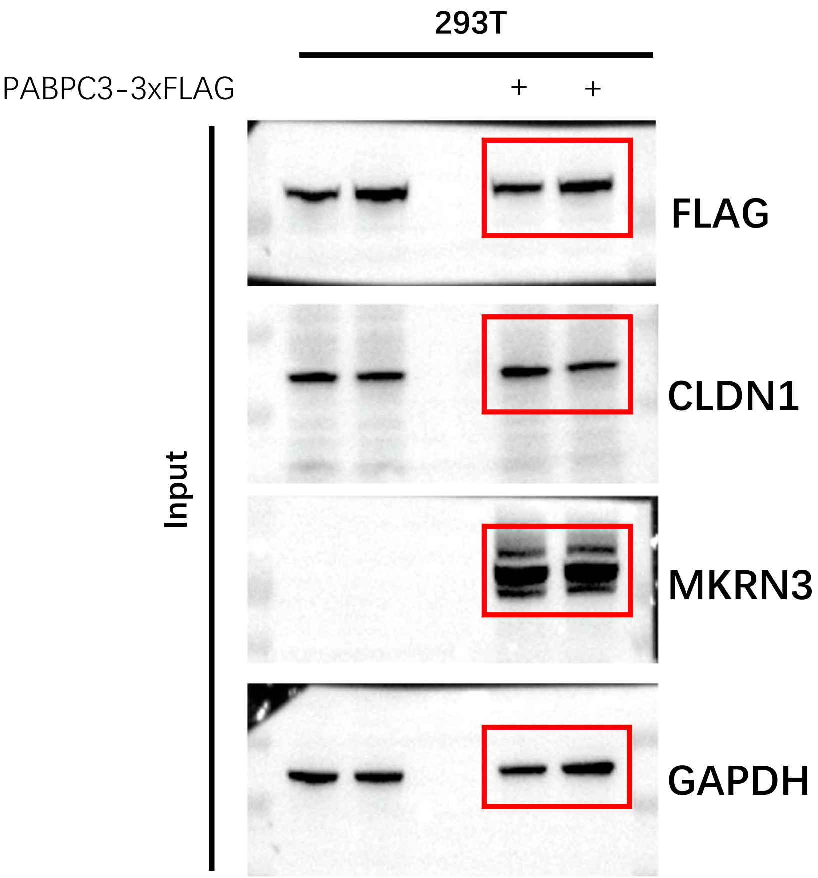

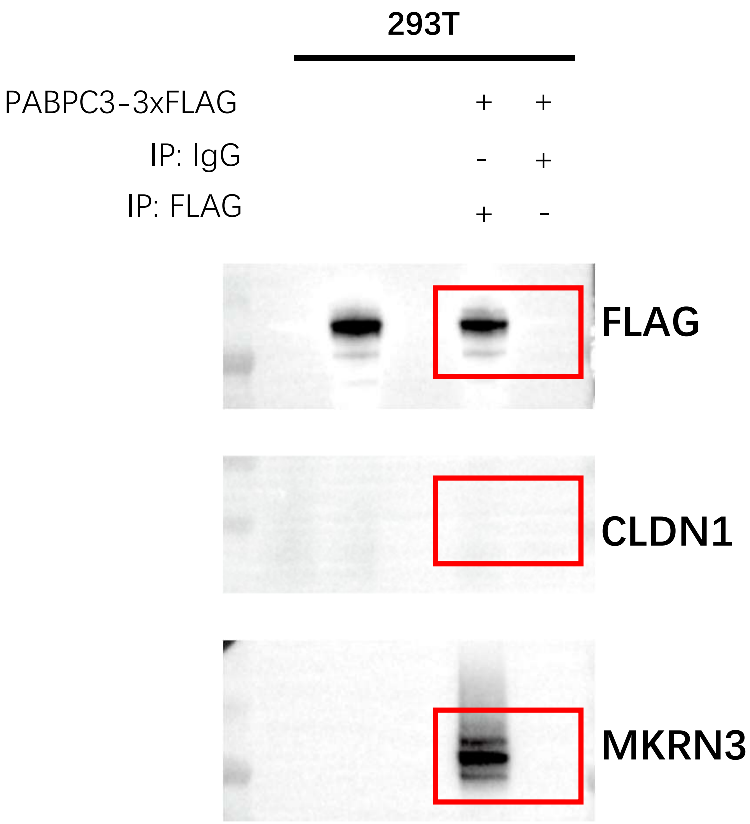

Full unedited gel for Supplementary Figure 1E
